# Nuclear DAB2IP regulates DNA replication initiation through activating PLK1-mediated HBO1 phosphorylation

**DOI:** 10.1093/nar/gkaf1179

**Published:** 2025-11-20

**Authors:** Zeng-Fu Shang, Lan Yu, Ciara Newman, Wei-Min Chen, Grant W Birdsong, Brett C Sharp, Michael D Story, Debabrata Saha, Anthony J Davis

**Affiliations:** Department of Radiation Oncology, University of Texas Southwestern Medical Center, Dallas TX 75390, United States; Department of Pathology, University of Texas Southwestern Medical Center, Dallas TX 75390, United States; Department of Radiation Oncology, University of Texas Southwestern Medical Center, Dallas TX 75390, United States; Department of Radiation Oncology, University of Texas Southwestern Medical Center, Dallas TX 75390, United States; Department of Radiation Oncology, University of Texas Southwestern Medical Center, Dallas TX 75390, United States; Department of Radiation Oncology, University of Texas Southwestern Medical Center, Dallas TX 75390, United States; Department of Radiation Oncology, University of Texas Southwestern Medical Center, Dallas TX 75390, United States; Department of Cancer Biology and Radiation Oncology, Mayo Clinic, Jacksonville, FL, United States; Department of Radiation Oncology, University of Texas Southwestern Medical Center, Dallas TX 75390, United States; Simmons Comprehensive Cancer Center, University of Texas Southwestern Medical Center, Dallas, TX 75390, United States; Department of Radiation Oncology, University of Texas Southwestern Medical Center, Dallas TX 75390, United States; Simmons Comprehensive Cancer Center, University of Texas Southwestern Medical Center, Dallas, TX 75390, United States

## Abstract

DAB2IP (Disabled homolog 2 interacting protein), a recognized tumor suppressor, plays a pivotal role in regulating various oncogenic pathways. Our previous research demonstrated that DAB2IP functions as a cell cycle regulator by facilitating PLK1-mediated mitosis progression. Here, we elucidate a novel function of DAB2IP in promoting DNA replication origin firing. Mechanistically, we identified that DAB2IP localizes to the nucleus, where it interacts with the histone acetyltransferase HBO1 and enhances the HBO1–PLK1 interaction. DAB2IP facilitates PLK1-mediated phosphorylation of HBO1, which subsequently promotes HBO1-directed acetylation of histone 3 at lysine 14 (H3K14Ac). This modification enables the loading of the minichromosome maintenance protein (MCM) complex onto chromatin, thereby supporting DNA replication and maintaining genome integrity. Additionally, we found that ATR regulates CDK1-mediated phosphorylation of DAB2IP, and that this phosphorylation is essential for the formation and activation of the HBO1-PLK1 complex. Ablation of DAB2IP phosphorylation results in increased genomic instability due to incomplete replication of genomic DNA, as evidenced by the accumulation of anaphase ultrafine bridges and 53BP1 nuclear bodies in G1 phase of the cell cycle. In summary, our findings underscore the critical regulatory role of DAB2IP in DNA replication initiation and genomic stability maintenance, providing new insights into its function in cellular homeostasis.

## Graphical Abstract

**Graphical Abstract:**
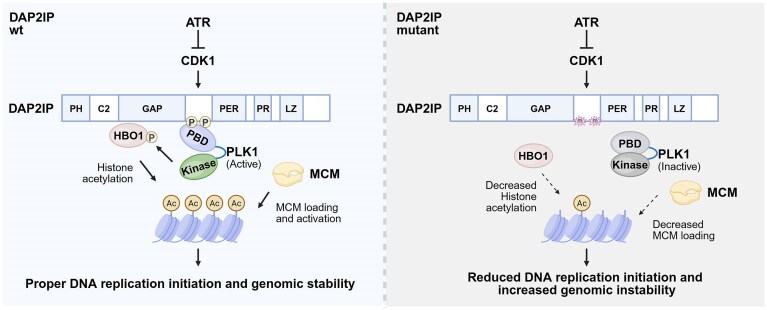

## Introduction

The tumor suppressor DAB2IP, also known as AIP1 (ASK1 interacting protein), was initially identified as a member of the RAS-GTPase activating protein (Ras-GAP) family [1, 2]. DAB2IP negatively regulates RAS-dependent mitogenic signaling via its Ras-GAP activity [2]. Additionally, DAB2IP acts as a scaffold to coordinate various signal transduction pathways involved in cell growth, apoptosis, and the epithelial-mesenchymal transition [3, 4]. For example, DAB2IP directly interacts with both the PI3K regulatory subunit (p85) and Akt, reducing the phosphorylation and activation of Akt [5]. Further, DAB2IP interacts with apoptosis signal-regulating kinase 1 (ASK1) and confers ASK1 activation and ASK1-mediated cell apoptosis [5]. DAB2IP also plays a role in the inflammatory response by interacting with TNF receptor-associated factor 2 (TRAF2) and inhibiting TNF-α-induced NF-κB activation [6]. Mutant p53 has been found to competitively bind to DAB2IP, leading to the reactivation of NF-κB and the rewiring of the inflammatory microenvironment within tumors [7, 8]. *DAB2IP* is often lost in the early stages of tumor development, resulting in the production of inflammatory mediators and an enrichment of macrophages, suggesting a potential role for DAB2IP in remodeling the tumor immune microenvironment [9]. Our previous research uncovered an additional function of DAB2IP, which is as a cell cycle regulator. We reported that DAB2IP was phosphorylated by CDK1, and this phosphorylated form of DAB2IP interacts with and activates the mitotic regulator, polo-like kinase 1 (PLK1) [10, 11]. The loss of DAB2IP expression or the inhibition of its CDK1-mediated phosphorylation causes significant mitotic abnormalities, including compromised kinetochore-microtubule attachment, impaired spindle assembly checkpoint, and subsequent chromosomal instability [10, 11]. Collectively, these findings highlight the multifaceted functions of DAB2IP in cellular processes, ranging from tumor suppression and signal transduction to cell cycle regulation and inflammation, underscoring its importance in maintaining cellular homeostasis.

The initiation of DNA replication in eukaryotic cells is a tightly regulated process, both temporally and spatially, ensuring that the genome is accurately duplicated once per cell cycle. This initiation begins with the association of the origin recognition complex (ORC) with replication origins on DNA during the late M phase [12]. ORC recruits Cdc6 and Cdt1, which are essential for the subsequent loading of the inactive double hexamer helicase, minichromosome maintenance complex (MCM 2–7), forming pre-replication complexes (pre-RCs). During the S phase, the replicative helicase complex is activated by the dual action of CDK and DDK kinases. This activation involves the phosphorylation of the pre-RCs and the recruitment of CDC45 and the GINS complex [13]. The chromatin environment is intricately linked with the temporal and spatial regulation of replication initiation. Genome-wide analysis revealed that pre-RC proteins are preferentially enriched in regions of low nucleosome occupancy [14]. Sequencing studies have shown that ORC selectively binds to open regions marked by active chromatin features, such as histone acetylation, the presence of the histone variant H3.3, and the recruitment of chromatin remodelers [15–17]. Accumulating evidence indicates that acetylation of histones H3 and H4 facilitates DNA replication initiation, likely by increasing the accessibility of chromatin [18]. It has been reported that acetylation of histone H4 at lysine 5, 8, and 12 by lysine acetyltransferase 7 (KAT7 or HBO1) contributes to chromatin loading of the MCM 2–7 complex [19]. HBO1 is also crucial for the acetylation of histone H3 at lysine 14 (H3K14ac), with genome-wide analyses revealing high enrichment of H3K14ac at ORC1-binding sites and replication origin regions [16]. The mitotic regulator PLK1 also plays a significant role in regulating DNA replication. PLK1 interacts with members of the MCM 2–7 complex [20, 21]. Additionally, PLK1 interacts with and phosphorylates HBO1, facilitating HBO1-mediated loading of the MCM 2–7 complex [22].

In this study, we identified a novel role of DAB2IP in regulating DNA replication initiation. We discovered that DAB2IP is located in the nucleus, where it interacts with the histone acetyltransferase HBO1. Nuclear DAB2IP facilitates the PLK1-mediated activation of HBO1, thereby regulating DNA replication by targeting histone H3K14 in the vicinity of the replication origin. Loss of DAB2IP results in reduced chromatin loading and phosphorylation of MCM2, leading to a decrease in replication initiation. Consequently, genomic instability increases, and cells undergo cell death due to incomplete replication of genomic DNA. These findings highlight an unexpected function of DAB2IP in the precise regulation of DNA replication, shedding new light on its role in maintaining genomic stability and cell viability.

## Materials and methods

### Cell culture, siRNA transfection, plasmids, and treatment

Human cervical cancer HeLa cells, human embryonic kidney 293T cells, human colon cancer HCT116 cells, and mouse embryonic fibroblasts (MEFs) were maintained in α-minimum essential medium (α-MEF, HyClone, Hudson, NH, USA), supplemented with 10% fetal bovine serum (FBS, HyClone, Hudson, NH, USA), 1 mM sodium bicarbonate, 100 U/mL penicillin, and 100 μg/mL streptomycin, 10 mM HEPES (HyClone, Hudson, NH, USA) in a humidified incubator at 37°C with 5% CO_2_. All cell lines were authenticated using short tandem repeat (STR) profiling detection by the Cell Bank of Typical Culture Preservation Committee of the Chinese Academy of Sciences. Cell lines were routinely tested using the Mycoplasma-free Mycoplasma Detection Kit (Beyotime, Shanghai, China).

Dr. Jer-Tsong Hsieh at UT Southwestern Medical Center kindly provided the primary MEFs utilized in this study. The MEFs used in this study were immortalized via expression of SV-40 antigen. HeLa cells were transfected with pGIPZDAB2IP-lentiviral-shRNAmir and pGIPZ-non-silencing-lentiviral shRNAmir [4] according to the manufacturer’s protocol. The cells were then selected by puromycin (ThermoFisher Scientific, Carlsbad, CA, USA) at a concentration of 0.5 g/mL for 3–4 weeks. Duplex siRNAs were synthesized (ThermoFisher Scientific, Carlsbad, CA, USA) based on experimentally validated target sequences for DAB2IP siRNA (5′-GGAGCGCAACAG UUACCUGTT-3′), siRNA2 (5′-GGUGAAGGACUUCCUGACATT-3′), or control siRNA (5′-CTGGACTTCC AGAAGAACA-3′). SiRNA was transfected into HeLa cells using Lipofectamine 3000 (ThermoFisher Scientific, Carlsbad, CA, USA).

The DAB2IP full-length expressing plasmids were cloned from human DAB2IP isoform 2 cDNA (1065 amino acids). The DAB2IP siRNA-resistant expression plasmid and various DAB2IP full-length and truncated expression plasmids were described previously [4, 10]. A sequence-verified open reading frame clone of PLK1 was cloned into pcDNA3.1 3xFLAG, pCMV-HA, and pcDNA5/FRT/TO V5 expression plasmids. A sequence-verified open reading frame clone of human HBO1 was cloned into the pcDNA3.1 3xFLAG expression plasmid. Point mutations for DAB2IP were generated by using a QuikChange II site-directed mutagenesis kit (Agilent, Santa Clara, CA, USA).

Cells were treated with 0.5 mM (MEFs) or 2 mM (HeLa cells) HU (Sigma, St. Louis, MO, USA), 5 μM CDK1 inhibitor RO-3306 (Selleckchem, Houston, TX, USA), 10 nM PLK1 inhibitor BI2536 (Sigma, St. Louis, MO, USA), or 5 μM ATR inhibitor VE-821 (Selleckchem, Houston, TX, USA) for the times indicated in the figure legends. For the colony survival experiment, MEFs were treated with 5, 10, 25, and 50 μM HU. HeLa cells were treated with 100 and 200 μM HU.

### Antibodies, immunoprecipitation, and immunofluorescence

The following primary antibodies were used in the present study: anti-DAB2IP, anti-MCM2, anti-MCM2-pSer40/41, anti-cyclin A (Bethyl Laboratories, Montgomery, TX, USA); anti-HBO1, anti-PARP, anti-cleaved caspase 3, anti-histone H3, anti-H3K14Ac, anti-p-SP motif, anti-p-TP motif, anti-53BP1 (Cell Signaling Technology, Danvers, MA, USA); anti-HA, control mouse IgG and rabbit IgG (Santa Cruz Biotechnology, Dallas, TX, USA); anti-α-tubulin, anti-V5, anti-FLAG (M2), anti-FLAG (M5), anti-β-actin (Sigma-Aldrich, St. Louis, MO, USA); anti-γH2AX, anti-BrdU/IdU (BD Biosciences, San Jose, CA, USA); anti-RPA2 (EMD Millipore, Temecula, CA, USA); anti-BrdU/CldU (Bio-Rad, Hercules, CA, USA), PLK1 (Abcam, Cambridge, MA, USA). The secondary antibodies used for immunofluorescence were Alexa Fluor 488 goat anti-mouse IgG, Alexa Fluor 488 goat anti-rabbit IgG, Alexa Fluor 488 goat anti-human IgG, Alexa Fluor 568 goat anti-mouse IgG, Texas red goat anti-rat IgG, and Alexa Fluor 568 goat anti-rabbit IgG (Invitrogen Carlsbad, CA, USA).

For immunoprecipitations, HeLa cell lysates were incubated overnight with anti-DAB2IP, anti-HBO1, or anti-FLAG (M2) antibodies and protein A/G sepharose. The sepharose beads were washed with lysate buffer three times and resuspended in a sodium dodecyl sulfate (SDS)-PAGE loading buffer for immunoblotting analysis using the indicated antibodies.

For the immunofluorescent staining experiments, cells with different treatment regimens were plated on 35 mm dishes with coverslips, fixed in 4% paraformaldehyde/PBS (phosphate-buffered saline) for 30 min, permeabilized in 0.5% Triton X-100/PBS for 15 min, and blocked in 5% bovine serum albumin (BSA)/PBS for 30 min. The samples were incubated with anti-γH2AX (1:1000), anti-53BP1 (1:1000), anti-RPA2 (1:500), anti-cyclin A (1:500), or anti-H3K14Ac (1:200) antibodies for 3 h at room temperature, washed three times in PBS (5 min each), and incubated with Alexa Fluor 488 and 568 secondary antibodies (1:1000) for 1 h. The cells were washed in PBS three times (5 min each) and mounted using VECTASHIELD with 4′, 6-diamidino-2-phenylindole (DAPI) (Vector Laboratories, Burlingame, CA, USA). Images were taken using a fluorescence microscope (Axio Imager M2, Carl Zeiss, Thornwood, NY, USA) and were recorded using AxioVision SE64 Rel.4.8 software (Carl Zeiss, Thornwood, NY, USA). For chromatin-bound RPA2 staining, cells were pre-extracted with CSK-T buffer (10 mM Pipes, pH 7.0, 100 mM NaCl, 300 mM sucrose, and 3 mM MgCl_2_, containing 0.5% Triton X-100) for 5 min before fixation.

### Clonogenic survival and cell proliferation assays

For the clonogenic survival assays, 400 or 800 *DAB2IP^+/+^* and *DAB2IP^−/−^* MEFs were plated on 60-mm dishes and allowed to grow for 10 days. Subsequently, the colonies were fixed and stained using a solution of 100% ethanol with 0.1% crystal violet. Colonies (≥50 cells) were scored, and the mean value for triplicate culture dishes was determined. For HU treatment, HeLa cells were transfected with siRNA to suppress *DAB2IP* expression or co-transfected with siRNA and siRNA-resistant constructs encoding wild-type DAB2IP (rWT), phosphorylation-deficient mutant DAB2IP-2A (r2A), or phosphorylation-mimetic mutant DAB2IP-2D (r2D). These cells, along with *DAB2IP^+/+^* and *DAB2IP^−/−^* MEFs, were plated in 60-mm dishes. Cells were treated with the indicated dose of HU for 10 days. Colonies (≥50 cells) were scored, and the mean value for triplicate culture dishes was determined. Cell survival was normalized to the plating efficiency of untreated controls for each cell type.

For cell proliferation/viability assays, 1000 cells were plated in 96-well microplates. Cell numbers were analyzed by using Cytation 5 Multi-Mode Reader (BioTek, Winooski, VT, USA) at the indicated days after plating.

### Proximity ligation assay

Exponentially growing HeLa cells stably expressing FLAG-DAB2IP or FLAG-VC, along with transient or stable *DAB2IP*-knockdown and control HeLa cells, as well as wild-type and *DAB2IP^−/−^* MEFs, were cultured on microscope slides overnight. Cells were treated with or without 5 µM ATR inhibitor (VE821) for 2 h as indicated in the figure legends. Proximity ligation assay (PLA) was performed using Duolink PLA reagents (Sigma-Aldrich) according to the manufacturer’s instructions and our previously published study [23]. Briefly, cells were fixed with 4% PFA/PBS for 20 min, treated with 0.5% Triton X-100/PBS for 15 min, and blocked with Duolink *in situ* blocking buffer for 1 h at room temperature. Cells were incubated with primary antibodies in Duolink *in situ* antibody diluent for 2 h, then washed with PBS twice. Cells were incubated with oligonucleotides-conjugated secondary antibodies (PLA probe anti-rabbit PLUS and anti-mouse MINUS). Cells were exposed to ligation buffer and went through rolling circle amplification with a fluorophore-labeled oligonucleotide probe. Cells were mounted in Vectashield mounting medium with DAPI (Vector Laboratories) and visualized using a Zeiss AxioImager M2 microscope equipped with an EC Plan-Neofluar 40X/0.75 objective. Antibodies used in the PLA experiments were mouse anti-FLAG (M5) (Sigma 1:200), rabbit anti-HBO1 (Cell Signaling Technology 1:100), mouse anti-histone H3 (Cell Signaling Technology 1:200), and mouse anti-PLK1 (Abcam 1:100).

### FLAG-tagged protein purification

For purification of FLAG-tagged DAB2IP, DAB2IP-2A, DAB2IP-2D, HBO1, and PLK1, the plasmids were transfected into HeLa cells. The cells expressing FLAG-DAB2IP, FLAG-DAB2IP-2A, and FLAG-DAB2IP-2D were treated with 5 μM VE-821 for 2 h before being harvested. FLAG-tagged protein purification was performed as described [24]. Cells were suspended briefly in lysis buffer 3 (50 mM HEPES 7.4, 500 mM NaCl, 0.5% NP-40, 10% Glycerol, 1 × phosphatase inhibitor cocktail, 1 × protease inhibitor cocktail) and sonicated on ice. Cell lysates were incubated with M2-FLAG beads (Sigma, St. Louis, MO, USA). FLAG-tagged HBO1 and PLK1 were treated with lambda phosphatase (γPP) (Sigma, St. Louis, MO, USA). Protein-bound M2-FLAG beads were washed with washing buffer 3 (50 mM HEPES pH 7.4, 1 M NaCl, 0.5% NP-40, 10% Glycerol, 1 × phosphatase inhibitor cocktail, 1 × protease inhibitor cocktail), lysis buffer 3, washing buffer 4 (50 mM HEPES 7.4, 150 mM NaCl, 0.5% NP-40, 10% Glycerol), washing buffer 5 (50 mM HEPES 7.4, 150 mM NaCl, 10% Glycerol), and washing buffer 6 (50 mM HEPES 7.4, 80 mM KCl, 10% Glycerol). Finally, proteins were eluted with washing buffer 6 containing 250 µg/mL 3 × FLAG peptides. Protein purity was examined by Coomassie blue gel staining after SDS-PAGE, and the concentration was measured with Bio-Rad Protein Assay Kit 1.

### In vitro kinase assay

For the *in vitro* PLK1 kinase assay, purified FLAG-PLK1 was incubated with FLAG-DAB2IP, FLAG-DAB2IP-2A, or FLAG-DAB2IP-2D in PLK1 kinase reaction buffer [20 mM HEPES (pH 7.4), 100 mM KCl, 10 mM MgCl2, 1 mM EDTA, 0.5 mM DTT, 5% glycerol, 10 M ATP, and 0.17 μM γ-32P ATP] with FLAG-HBO1 as substrates. The reaction was incubated for 30 min at 30°C and was stopped by adding sodium dodecyl sulfate (SDS) sample buffer. After the kinase reaction, samples were subject to immunoblotting analysis using an anti-p-S-P antibody.

### Chromatin fractions isolation (CSK extraction method)

*DAB2IP^+/+^* and *DAB2IP^−/−^* MEFs were harvested and incubated with CSK-100 buffer (100 mM NaCl, 300 mM sucrose, 3 mM MgCl2, 10 mM PIPES, pH 6.8, 1 mM EGTA, 0.2% Triton X-100) containing protease inhibitors at 4 °C for 15 min. Cell pellets were spun down and washed one time using CSK buffer. These pellets were incubated with lysis buffer (50 mM HEPES, pH 7.5, 50 mM NaCl, 0.05% SDS, 2 mM MgCl2, 10% Glycerol, 0.1% Triton X-100, 10 units of Benzonase nuclease) containing protease inhibitors at 4 °C overnight. The chromatin-associated proteins were released into supernatants.

### Subcellular fractionation

The association of the MCM complex and HBO1 with chromatin in *DAB2IP*-proficient or -deficient cells (*DAB2IP^+/+^* and *DAB2IP^−/−^* MEFs, or control and siRNA-mediated knockdown HeLa cells) was examined using the Thermo Fisher Subcellular Protein Fractionation Kit as described [25]. Briefly, the *DAB2IP^+/+^, DAB2IP^−/−^* MEFs, or control or siRNA-mediated knockdown HeLa cells were mock-treated or treated with the indicated dose of HU for 2 h. Next, the cells were harvested after trypsinization and processed with the Thermo Fisher Subcellular Protein Fractionation Kit according to the manufacturer’s instructions. The protein concentration of each sample was measured using a Pierce BCA Protein Assay kit (Thermo Fisher), and 30 μg protein of each sample was separated via SDS-PAGE. The indicated protein was determined by immunoblotting analysis.

### Chromatin flow cytometry

Chromatin-bound MCM2 was examined using flow cytometry as previously described [26–28]. Briefly, cells were harvested with trypsin and resuspended as single cells, washed with PBS, and then incubated with CSK-T for 15 min on ice. Next, 1% BSA/PBS was added and mixed, and then the cells were washed one time with 1% BSA/PBS and then fixed in 4% PFA/PBS for 20 min at room temperature. Fixed cells were washed one time with 1% BSA/PBS and blocked in 1% BSA/PBS for 20 min. The primary antibody against MCM2 and goat anti-rabbit IgG cross-adsorbed secondary antibody (Alexa Fluor TM 488, A110088, Thermo Fisher) were sequentially labelled. DNA content was stained with 10 µg/mL of propidium iodide in PBS. RNA was digested by 0.5 µg/mL RNase A.

### Chromosome spread assays

MEFs were treated with 0.5 mM HU for 2 h. After 6 h of treatment with 100 ng/mL colcemid (Irvine Scientific, Santa Ana, CA, USA), cells were collected and hypotonically swollen in pre-warmed 75 mM KCl for 13 min at 37°C. Cells were fixed in freshly made Carnoys fixative solution (methanol: acetic acid 3:1) for 2–3 times. Cells were dropped onto warmed glass slides and dried overnight. Slides were stained with 5% Giemsa (Sigma, St. Louis, MO, USA) for 10 min at room temperature, gently rinsed with running water, air dried, and mounted. Slides were visualized using a microscope (Axio Imager M2, Carl Zeiss, Oberkochen, Germany) and were recorded with AxioVision SE64 Rel.4.8 software (Carl Zeiss, Oberkochen, Germany).

### DNA fiber assay

The DNA replication progression was examined using the DNA fiber assay as described previously [23]. Cells were labeled sequentially with 100 μM iododeoxyuridine (IdU) for 10 min and chlorodeoxyuridine (CldU) for 20 min. DNA fibers were spread as described [29] and stained with primary antibodies (mouse anti-BrdU/IdU, IgG1, BD, #347 580, NJ, USA, and rat anti-BrdU/CldU, abcam, ab6326, Cambridge, UK) and fluorescence conjugated secondary antibodies (Alexa Fluor 488 anti-rat and Texas Red anti-mouse, Thermo Fisher Scientific, MA, USA). Single DNA fibers are visualized by an anti-ssDNA antibody (mouse anti-single strand DNA, IgG2a, Millipore #MAB3034, MA, USA). Fibers were imaged using the Zeiss AxioImager M2 and measured using the AxioVision software (x64 version 4.9.1).

### Statistical analysis

Wilcoxon rank-sum test for comparisons between two groups and one-way ANOVA for comparisons among more than two independent groups were performed to assess statistical significance. All statistical analyses were performed using the Prism GraphPad software. Statistical significance was defined as *P < 0.05, and values of **P < 0.01, ***P < 0.001 were also shown in different experiments.

## Results

### DAB2IP depletion decreases DNA replication initiation

**Figure 1.**
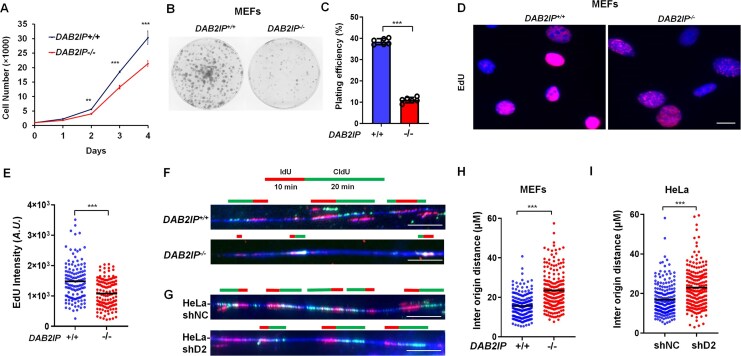
DAB2IP loss decreases DNA replication initiation. (**A**) Growth curve of transformed *DAB2IP^+/+^* and *DAB2IP^−/−^* mouse embryonic fibroblasts (MEFs). Cell numbers were analyzed using a cell counter (Beckman Coulter Z2). Data are presented as mean ± s.d. from six independent experiments. One-way ANOVA test was performed to assess statistical significance, **P < 0.01; ***P < 0.001. (**B**) Transformed *DAB2IP^+/+^* and *DAB2IP^−/−^* MEFs were plated at the indicated cell number, and colony formation was assessed. (**C**) The plating efficiency was counted in each group. Data are presented as mean ± s.d. from 6 independent experiments. The Wilcoxon rank-sum test was performed to assess statistical significance, ***P < 0.001. (**D**) and (**E**) *DAB2IP^+/+^* and *DAB2IP^−/−^* MEFs were pulse-labeled with 50 μM EdU for 30 min, and EdU labeling was assessed via microscopy. Data are presented as EdU densities in nuclei (*n* > 100). The horizontal bars represent the mean of each group. The Wilcoxon rank-sum test was used to examine statistical significance, ***P < 0.001. Scale bar = 10 μm. (**F-I**) Representative images (**F**) of DNA fibers in *DAB2IP^+/+^* and *DAB2IP^−/−^* MEFs after sequential labeling with iododeoxyuridine (IdU, 10 min) and chlorodeoxyuridine (CldU, 20 min). The inter-origin distance (**H**) of *DAB2IP^+/+^* and *DAB2IP^−/−^* MEFs is quantified. *n* > 200 from three independent experiments. The horizontal bars represent the mean of each group. The Wilcoxon rank-sum test was used to examine statistical significance, ***P < 0.001. Representative images (**G**) of DNA fibers were analyzed in *DAB2IP*-knocked-down and control (shNC) HeLa cells after sequential labeling with IdU (10 min) and CldU (20 min). The inter-origin distance (**I**) is quantified. *n* > 200 from three independent experiments. The horizontal bars represent the mean of each group. The Wilcoxon rank-sum test was used to examine statistical significance, ***P < 0.001

In our previous study, we demonstrated that DAB2IP modulates mitosis regulation [10, 11]. Here, we investigated the impact of DAB2IP on DNA replication progression. Initially, we used mouse embryonic fibroblasts (MEFs) from *DAB2IP^+/+^* and *DAB2IP^−/−^* mice. Consistent with DAB2IP’s role in cell cycle progression, we observed a decreased growth rate in *DAB2IP^−/−^* MEFs compared to *DAB2IP^+/+^* MEFs, as evidenced by proliferation and colony formation assays (Fig. 1A–C). To delve deeper, we examined DNA synthesis by assessing the incorporation of 5-ethynyl-2′-deoxyuridine (EdU) in *DAB2IP^+/+^* and *DAB2IP^−/−^* MEFs. Our results revealed reduced EdU incorporation in *DAB2IP^−/−^* MEFs compared to *DAB2IP^+/+^* MEFs, indicating impaired DNA synthesis upon DAB2IP loss (Fig. 1D and E). To confirm that DAB2IP regulates DNA synthesis, we knocked down DAB2IP in the cervical cancer line HeLa cells and then assessed EdU incorporation. Consistently, the data revealed that loss of DAB2IP attenuates EdU incorporation compared to control cells (Supplementary Fig. S1A and B). The decreased DNA synthesis could be attributed to a reduction in the frequency of DNA replication origin activation or a decrease in DNA replication fork progression speed, or both. To elucidate the details of the DNA replication, we employed DNA fiber assays by sequentially pulse-labeling cells with iododeoxyuridine (IdU) and chlorodeoxyuridine (CIdU). Our findings demonstrated a significant increase in the distance between two DNA replication origins in *DAB2IP*-knockout MEFs compared to wild-type MEFs, suggesting a decreased frequency of origin firing during DNA replication initiation (Fig. 1F and H). Additionally, we observed an increased inter-origin distance in *DAB2IP*-knockdown HeLa cells compared to HeLa control cells (Fig. 1G and I). Due to hypermethylation of the DAB2IP gene promoter, prostate cancer C4-2 cells exhibit down-regulated endogenous DAB2IP expression [30]. Consistently, an increase in the distance between two DNA replication origins was observed in C4-2 control cells (C4-2 Neo) compared to C4-2 cells that stably express DAB2IP (C4-[2] D2) (Supplementary Fig. S1E). We also observed that the progression of ongoing DNA replication tracks is markedly attenuated in *DAB2IP^−/−^* MEFs compared to *DAB2IP^+/+^* MEFs (Supplementary Fig. S1C). Conversely, stable expression of DAB2IP promoted replication tracks speed in C4-2 cells (Supplementary Fig. S1F). However, replication tracks exhibited a slight increase in stably DAB2IP-depleted HeLa cells (Supplementary Fig. S1D), suggesting a compensatory mechanism to support replication in some cells [31]. Our studies suggest that DAB2IP plays a critical role in facilitating normal DNA replication initiation.

### Loss of DAB2IP exacerbates DNA replication stress and genomic instability

**Figure 2.**
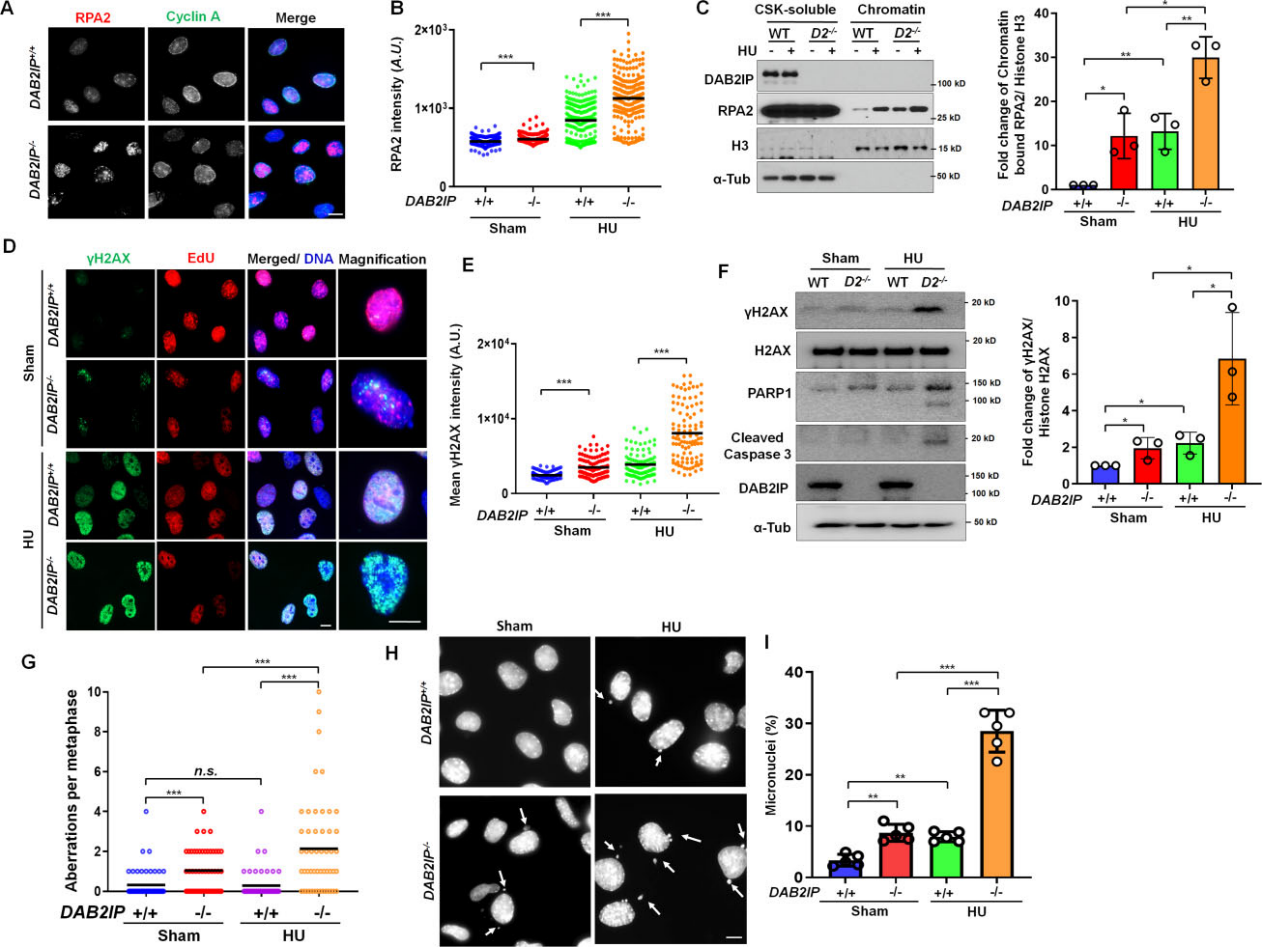
Loss of DAB2IP causes DNA damage and genomic instability. (**A**) and (**B**) *DAB2IP^+/+^* and *DAB2IP^−/−^* MEFs were treated with 0.5 mM hydroxyurea (HU) for 2 h, pre-extracted with CSK buffer, and stained using RPA2 and Cyclin A antibodies. Data are presented as RPA2 densities in Cyclin A^+^ nuclei (*n* > 100). The horizontal bars represent the mean of each group. One-way ANOVA test was used to examine statistical significance, ***P < 0.001. Scale bar = 10 μm. (**C**) *DAB2IP^+/+^* and *DAB2IP^−/−^* MEFs were treated with 0.5 mM HU for 2 h. Subsequently, CSK buffer soluble and chromatin fractions were isolated for immunoblotting to examine the recruitment of indicated proteins after HU treatment. Right panel: The chromatin RPA2 was normalized to the total histone H3 protein level, then graphed for three independent experiments. Data are presented as mean ± s.d. from three independent experiments. One-way ANOVA test was performed to assess statistical significance, *P < 0.05, **P < 0.01. (**D**) and (**E**) Loss of DAB2IP results in increased DNA DSBs formation in normal conditions and after treatment with HU. Representative images of γH2AX (Green) and EdU (Red) in *DAB2IP^+/+^* and *DAB2IP^−/−^* MEFs in the presence or absence of HU treatment. Data were quantified as γH2AX intensities (*n* > 100 from three independent experiments). The horizontal bars represent the mean of each group. One-way ANOVA test was used to examine statistical significance, ***P < 0.001. Scale bar = 10 μm. (**F**) Deletion of DAB2IP induces DSBs and apoptosis in normal cycling cells and after treatment with HU. Cells were exposed to 0.5 mM of HU for 2 h and subjected to immunoblotting with anti-PARP, anti-cleaved caspase 3, anti-DAB2IP, and anti-α-Tubulin antibodies at 24 h after HU treatment. Right panel: The γH2AX was normalized to the total H2AX protein level, then graphed for three independent experiments. Data are presented as mean ± s.d. from three independent experiments. One-way ANOVA test was performed to assess statistical significance, *P < 0.05. (**G**) Deletion of DAB2IP results in increased spontaneous and HU-induced chromosome aberrations compared to wild-type cells. The number of aberrant chromosomes in each cell was quantified in normal cycling cells and after HU treatment (*n* > 100 from three independent experiments). The horizontal bars represent the mean of each group. One-way ANOVA test was used to examine statistical significance, ***P < 0.001; “*n.s*.” = no significance. (**H**) and (I) Representative images (**H**) of micronuclei in different groups. (**I**) The percentage of cells with micronuclei was quantified. Data are presented as mean ± s.d. from five independent experiments. One-way ANOVA test was performed to assess statistical significance, **P < 0.01; ***P < 0.001. Scale bar = 10 μm.

Given that compromised DNA replication can lead to replication stress, we next investigated the overall levels and chromatin binding of the single-stranded DNA (ssDNA) association protein RPA in untreated cycling cells and after induction of replication stress via hydroxyurea (HU) treatment [23, 32]. In *DAB2IP^−/−^* MEFs, chromatin-bound RPA2 was elevated both in the absence and presence of HU compared to *DAB2IP^+/+^* MEFs (Fig. 2A–C). This increase in RPA2 intensity under both conditions was also observed in HeLa cells with *DAB2IP* knockdown compared to control cells (Supplementary Fig. S1G and H). Additionally, increased ssDNA formation as monitored by BrdU labeling was observed in *DAB2IP^−/−^* MEFs compared to *DAB2IP^+/+^* MEFs under both basal conditions and after HU treatment, further supporting the notion that DAB2IP loss results in replication stress (Supplementary Fig. S1 I and J). Since increased replication stress can lead to the induction of DNA double-strand breaks (DSBs) and genomic instability, we subsequently assessed these factors in *DAB2IP^−/−^* MEFs. We found that *DAB2IP^−/−^* MEFs exhibited more intense staining for the DSB marker γH2AX compared to *DAB2IP^+/+^* MEFs, both in the absence and presence of HU treatment (Fig. 2D and E). Immunoblotting confirmed that γH2AX levels were increased, and this correlated with increased apoptosis as assessed by cleaved caspase 3 and PARP1 cleavage in *DAB2IP^−/−^* MEFs compared to *DAB2IP^+/+^* MEFs in both normal cycling cells and after induction of exogenously induced replication stress (Fig. 2F). Additionally, *DAB2IP^−/−^* MEFs displayed a higher frequency of chromosomal aberrations and increased incidence of micronuclei in both the absence and presence of HU compared to *DAB2IP^+/+^* MEFs (Fig. 2G–I). Finally, the loss of DAB2IP in MEFs, HeLa, and HCT116 cells resulted in increased sensitivity to HU (Supplementary Fig. S1K–M). Collectively, these findings underscore the critical role of DAB2IP in promoting DNA replication and maintaining genomic stability.

### DAB2IP interacts with HBO1 in the nucleus and promotes chromatin loading of HBO1

**Figure 3.**
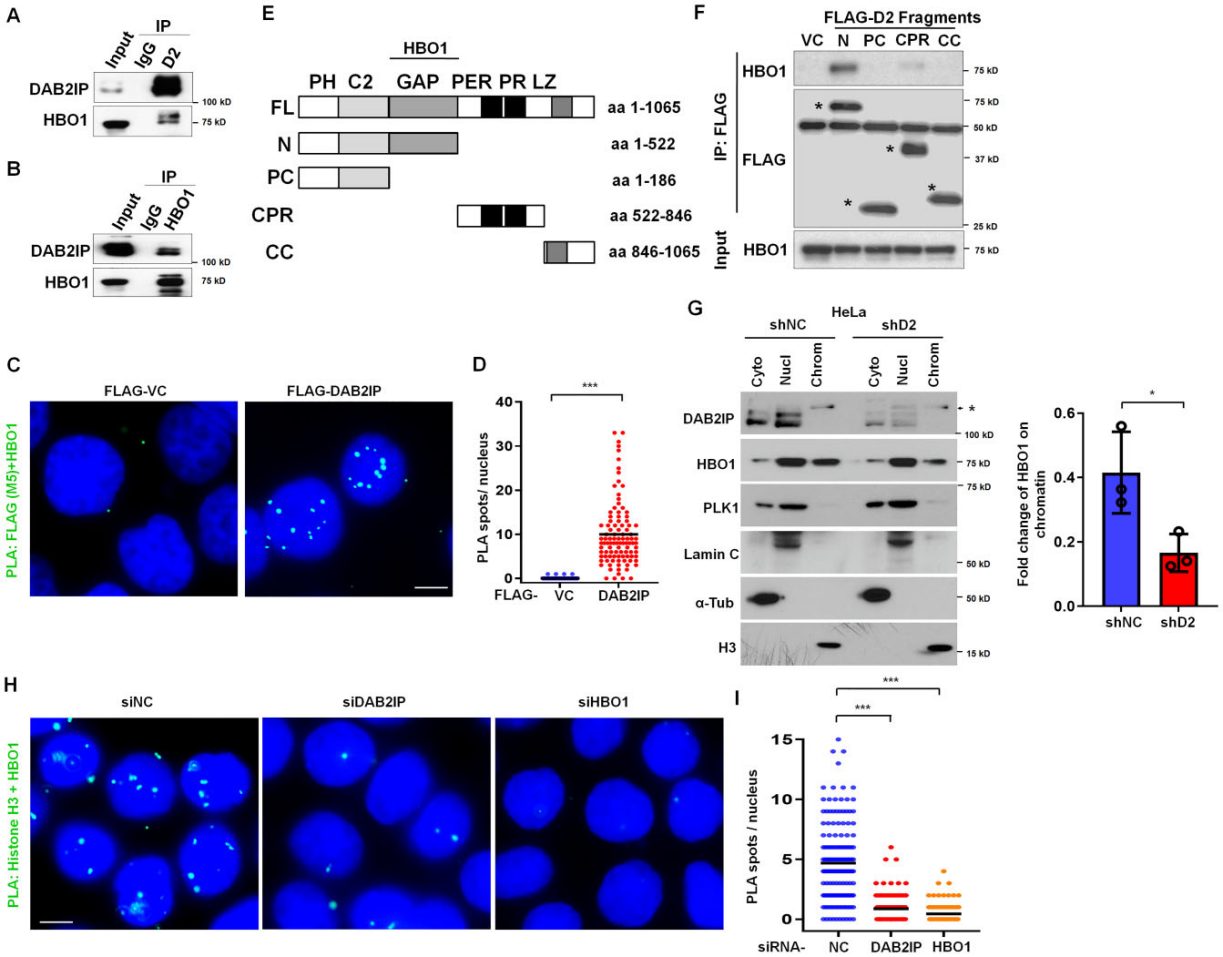
DAB2IP interacts with HBO1 and facilitates the chromatin binding of HBO1. (**A**) and (**B**) HeLa cell lysates were immunoprecipitated with anti-DAB2IP (**A**), anti-HBO1 (**B**), or control IgG antibodies, and the ability to co-immunoprecipitate the other protein was assessed by immunoblotting. (**C**) and (**D**) FLAG-DAB2IP and FLAG-VC stably expressed HeLa cells were fixed for PLA staining (green) using anti-FLAG (M5) and anti-HBO1 antibodies. Representative images (**C**) of PLA foci in both groups. Scale bar = 10 μm. (**D**) The quantification of PLA spots per nucleus in FLAG-DAB2IP and FLAG-VC stably expressed HeLa cells. *n* > 100 from three independent experiments. The horizontal bars represent the mean of each group. The Wilcoxon rank-sum test was used to examine statistical significance, ***P < 0.001. (**E**) Schema of different truncations of DAB2IP. (**F**) FLAG-tagged DAB2IP truncation proteins were transiently expressed in HeLa cells, they were immunoprecipitated using FLAG antibodies, and their ability to interact with HBO1 was assessed via immunoblotting. (**G**) Cytosol (Cyto), soluble nuclear (Nucl), and chromatin-enriched (Chrom) fractions were isolated from DAB2IP-depleted and control HeLa cells for immunoblotting to assess the sub-cellular localization of indicated proteins. * represents a non-specific signal. Right panel: The chromatin-associated HBO1 was normalized to the total nuclear HBO1 protein level, then graphed for three independent experiments. Data are presented as mean ± s.d. from three independent experiments. The Wilcoxon rank-sum test was performed to assess statistical significance, *P < 0.05. (**H**) and (I) HeLa cells were transfected with siRNA against *DAB2IP, HBO1*, and a non-specific control for 48 h and were fixed for PLA staining (green) using anti-histone H3 and anti-HBO1 antibodies. Representative images (**H**) of PLA foci in different groups. Scale bar = 10 μm. (**I**) Quantification of PLA spots per nucleus in DAB2IP-, HBO1-depletion, and control HeLa cells. *n* > 100 from three independent experiments. The horizontal bars represent the mean of each group. One-way ANOVA test was used to examine statistical significance, ***P < 0.001.

We next aimed to elucidate the regulatory role of DAB2IP in DNA replication progression. Utilizing co-immunoprecipitation followed by mass spectrometry (co-IP-MS) analysis, we identified that DAB2IP interacts with the histone acetyltransferase HBO1, a facilitator of DNA replication initiation. To determine whether DAB2IP directly interacts with HBO1, we performed reciprocal co-IP experiments, which confirmed a protein-protein interaction between DAB2IP and HBO1 (Fig. 3A and B). Additionally, we observed that endogenous HBO1 interacts with transiently expressed FLAG-tagged DAB2IP (Supplementary Fig. S2A). This interaction is further corroborated by *in situ* PLA, which showed that stably expressed FLAG-DAB2IP, but not FLAG-tag alone, associates with HBO1 in the nucleus (Fig. 3C and D). To delineate the region of DAB2IP responsible for its interaction with HBO1, we transiently expressed FLAG-tagged truncations of DAB2IP in HeLa cells, performed immunoprecipitation using an anti-FLAG antibody, and assessed the interaction with endogenous HBO1. Our data revealed that the GAP domain of DAB2IP preferentially interacts with endogenous HBO1 (Fig. 3E and F). Notably, DAB2IP predominantly resides in the cytoplasm, where it modulates oncogenic pathways, but our data indicated that DAB2IP may function in the nucleus [1]. To verify the nuclear localization of DAB2IP, we conducted subcellular fractionation assays, revealing its presence in the nuclear fraction of HeLa cells, DAB2IP-complemented C4-2 cells, and MEFs (Fig. 3G and Supplementary Fig. S2B and C). We identified the sequence, ^157^KKKKK^161^, as a probable nuclear localization signal (NLS) of DAB2IP. However, mutation of this sequence did not affect the nuclear localization of DAB2IP (Supplementary Fig. S2D). Both N- and C-terminal truncations retained the capability for nuclear localization, indicating the presence of multiple, non-classical NLSs within DAB2IP (Supplementary Fig. S2D). Although DAB2IP recruitment to chromatin was not observed, HBO1 chromatin binding was significantly reduced in *DAB2IP*-deficient cells (Fig. 3G, Supplementary Fig. S2B and C). Given HBO1’s essential role in histone H3 acetylation, we performed PLA using antibodies against histone H3 and HBO1 in DAB2IP and HBO1 knockdown HeLa cells. Knockdown of either DAB2IP or HBO1 led to a reduced number of PLA foci in the nucleus (Fig. 3H and I, Supplementary Fig. S2E), suggesting that DAB2IP facilitates HBO1 chromatin binding and its recruitment to histone H3. This observation was further supported by data from *DAB2IP*-KO MEFs, where histone H3-HBO1 foci were significantly reduced compared to wild-type MEFs (Supplementary Fig. S2F and G). Our findings suggest that DAB2IP plays a crucial role in facilitating HBO1 chromatin binding and its recruitment to histone H3, thereby influencing DNA replication initiation.

### Loss of DAB2IP leads to reduced H3K14 acetylation, impairment of MCM chromatin-binding, and decreased MCM2 phosphorylation

**Figure 4.**
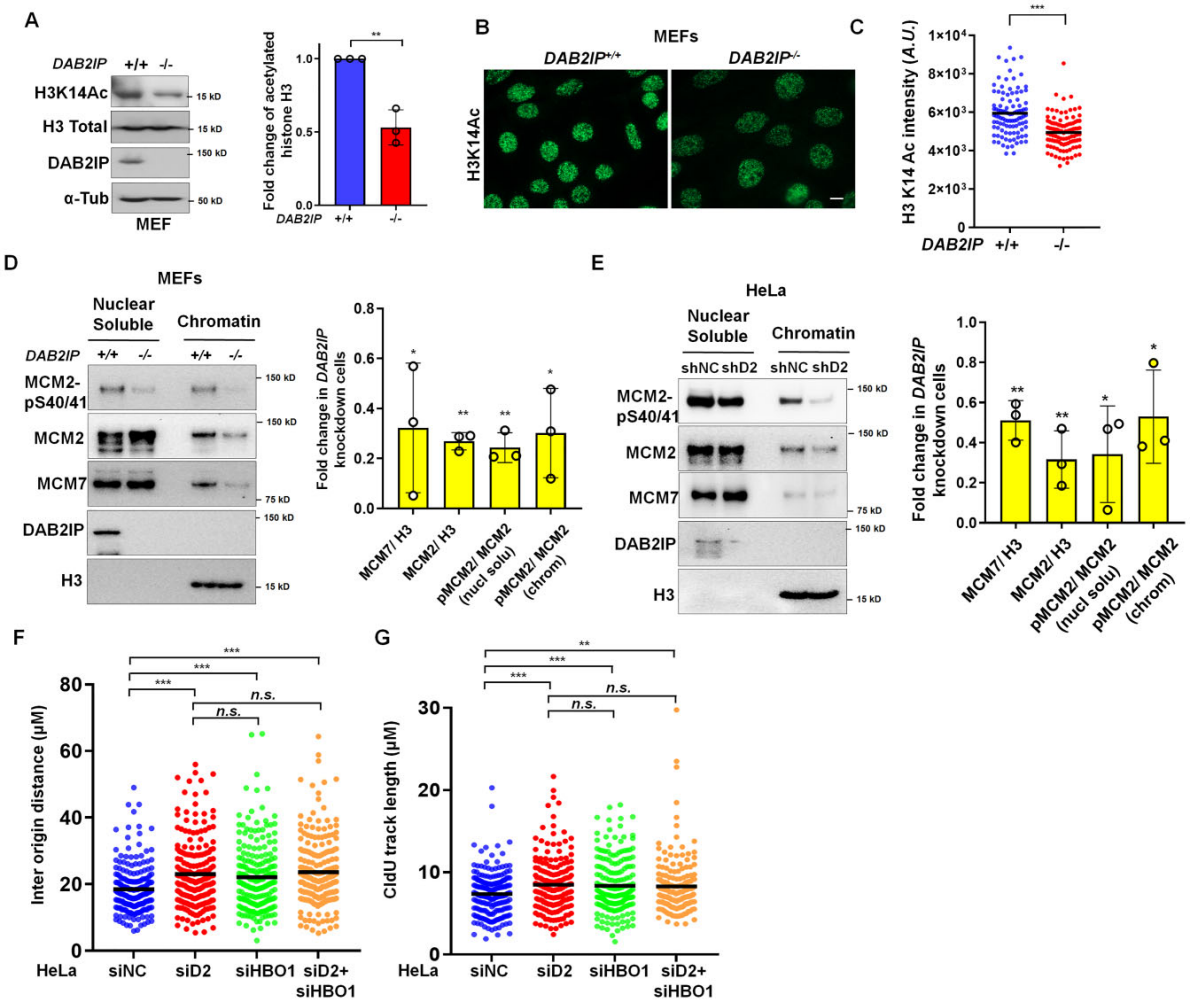
DAB2IP promotes HBO1-mediated H3K14 acetylation and assembly of the MCM complex on chromatin. (**A**) Acetylation of the histone H3 at lysine K14 (H3K14Ac) is decreased in DAB2I*P^−/−^* MEFs. Immunoblot analysis of indicated proteins in *DAB2IP^+/+^* and DAB2I*P^−/−^* MEFs. Right panel: The acetylated histone H3 at K14 site was normalized to the total histone H3 protein level, then the fold change was normalized to the *DAB2IP^+/+^* cells and graphed for three independent experiments. Data are presented as mean ± s.d. from three independent experiments. The Wilcoxon rank-sum test was performed to assess statistical significance, **P < 0.01. (**B**) and (**C**) Immunofluorescent staining of H3K14 acetylation in *DAB2IP^+/+^* and DAB2I*P^−/−^* MEFs. Quantification of H3K14Ac intensities was performed (*n* > 100 from three independent experiments). The horizontal bars represent the mean of each group. The Wilcoxon rank-sum test was used to examine statistical significance, ***P < 0.001. Scale bar = 10 μm. (**D**) The soluble nuclear and chromatin fractions of *DAB2IP^+/+^* and DAB2I*P^−/−^* MEFs were isolated for immunoblotting to examine the amount of MCM2, MCM7, and phosphorylated MCM2-S40/41 in each fraction. Right panel: The chromatin MCM2 and MCM7 were normalized to the chromatin histone H3 protein level, and the phosphorylated MCM2-Ser40/41 in soluble nucleus and chromatin were normalized to MCM2 protein in each fraction, and then graphed for three independent experiments. Data are presented as mean ± s.d. from three independent experiments. Wilcoxon rank-sum test was performed to assess statistical significance, *P < 0.05, **P < 0.01. (**E**) The soluble nuclear and chromatin fractions of *DAB2IP*-knocked-down and control (shNC) HeLa cells were isolated for immunoblotting to examine the amount of MCM2, MCM7, and phosphorylated MCM2-S40/41 in each fraction. Right panel: The chromatin MCM2 and MCM7 were normalized to the chromatin histone H3 protein level, and the phosphorylated MCM2-Ser40/41 in soluble nucleus and chromatin were normalized to MCM2 protein in each fraction, then graphed for three independent experiments. Data are presented as mean ± s.d. from three independent experiments. Wilcoxon rank-sum test was performed to assess statistical significance, *P < 0.05, **P < 0.01. (**F**) and (**G**) HeLa cells were transfected with siRNA against *DAB2IP, HBO1, D2*+ *HBO1*, and a non-specific control for 48 h. DNA fiber analysis was performed in the indicated groups of HeLa cells after sequential labeling with IdU (10 min) and CldU (20 min). The inter-origin distance (**F**) and the CIdU tracks length (**G**) are quantified. *n* > 200 from three independent experiments. The horizontal bars represent the mean of each group. One-way ANOVA test was used to examine statistical significance, “*n.s*.” = no significance; ***P < 0.001.

HBO1 is essential for the acetylation of histone 3 on lysine 14 (H3K14) [33]. Given that DAB2IP modulates HBO1 recruitment to the chromatin, we next investigated whether DAB2IP influences HBO1 activity. We observed that acetylation of H3K14 is reduced in *DAB2IP^−/−^* MEFs compared to *DAB2IP^+/+^* MEFs, as shown by immunoblotting and immunofluorescent staining (Fig. 4A–C), and that this decrease was not due to a loss in total histone H3 (Supplementary Fig. S2 H and I). Similarly, knockdown of DAB2IP in HeLa cells led to decreased H3K14 acetylation, which was restored upon re-expression of DAB2IP (Supplementary Fig. S2J and K). Considering the crucial role of HBO1 in DNA replication licensing, we assessed the impact of DAB2IP loss on this process by examining the loading of the MCM complex onto chromatin in *DAB2IP^−/−^* MEFs compared to *DAB2IP^+/+^* MEFs. The absence of DAB2IP significantly impaired the chromatin loading of MCM2 and MCM7 (Fig. 4D). A consistent reduction in chromatin-bound MCM complex was also observed in *DAB2IP*-depleted HeLa cells (Fig. 4E). Furthermore, we observed impaired MCM2 chromatin association in *DAB2IP*-knockdown HeLa cells relative to control cells as assessed by flow cytometry (Supplementary Fig. 2 L-N). Phosphorylation of MCM2 on serines 40 and 41 (S40/41) is pivotal for activating pre-replication complexes and facilitating replication fork progression [34, 35]. Therefore, we next assessed if DAB2IP modulates MCM2 phosphorylation. Our analysis showed a clear reduction in MCM2 phosphorylation at S40/41 in *DAB2IP^−/−^* MEFs compared to *DAB2IP^+/+^* MEFs, and in *DAB2IP* knockdown HeLa cells compared to control cells (Fig. 4D and E). Our findings indicate that the loss of DAB2IP leads to reduced H3K14 acetylation, impaired MCM chromatin binding, and decreased MCM2 phosphorylation. To further clarify the relationship between DAB2IP and HBO1 in DNA replication progression, we performed a DNA fiber assay in HeLa cells with transient knockdown of *HBO1* and *DAB2IP. DAB2IP*-depleted cells exhibited a significantly longer inter-tract distance compared to control cells, similar to the distance observed in HBO1-knockdown cells (Fig. 4F). Simultaneous knockdown of DAB2IP and HBO1 did not further increase the origin distance, supporting the idea that DAB2IP regulates DNA replication initiation in an HBO1-mediated manner. Additionally, transient knockdown of both DAB2IP and HBO1 enhanced the fork elongation rate, likely as part of a compensatory mechanism (Fig. 4G). Collectively, the results reveal that DAB2IP plays a critical role in modulating HBO1 activity, thereby influencing histone acetylation and DNA replication processes. The absence of DAB2IP leads to diminished H3K14 acetylation, impaired loading of the MCM complex onto chromatin, and decreased MCM2 phosphorylation. These findings highlight the significance of DAB2IP in maintaining proper DNA replication through its interaction with HBO1.

### DAB2IP bridges HBO1 and PLK1 and promotes PLK1-mediated phosphorylation of HBO1

**Figure 5.**
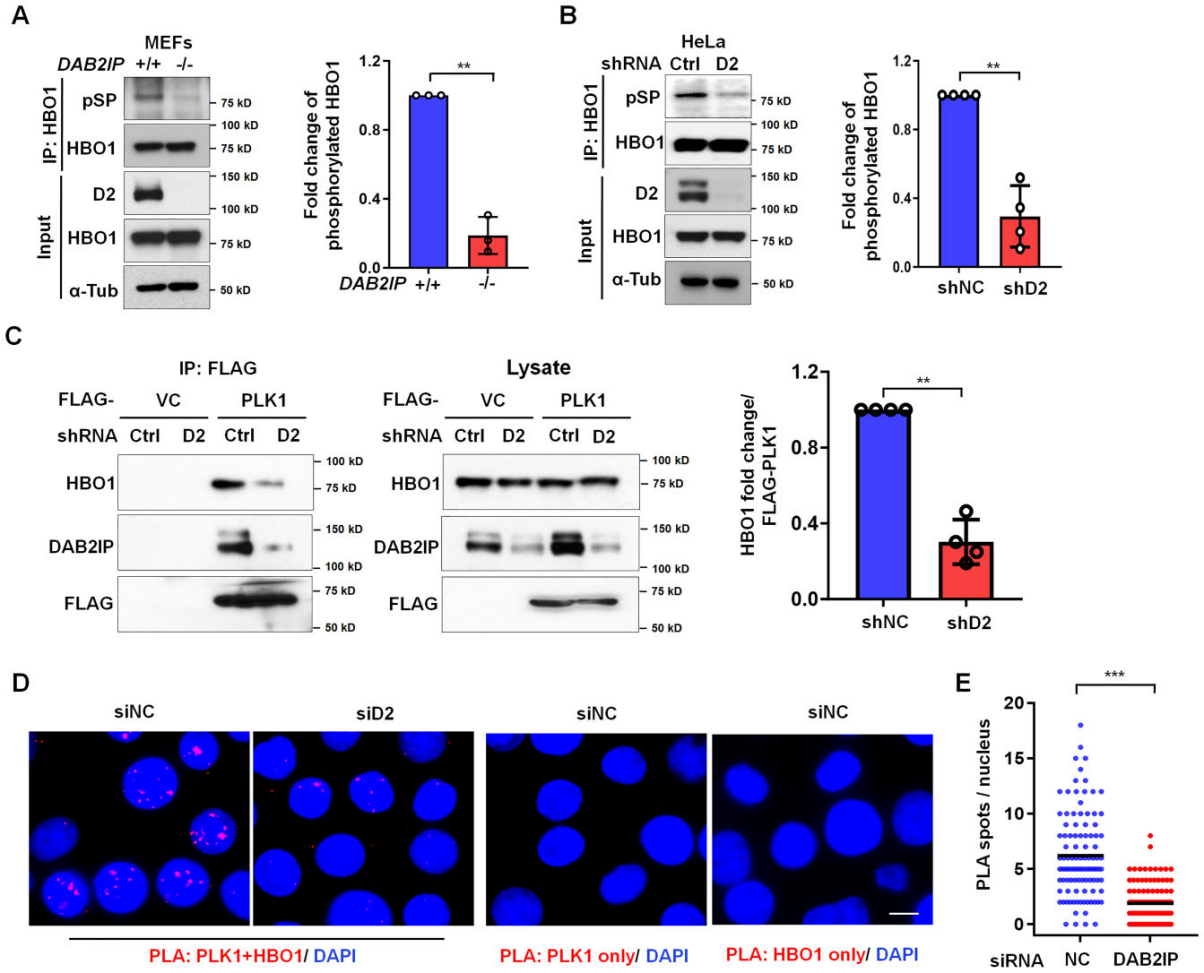
DAB2IP facilitates PLK1-HBO1 interaction and PLK1-mediated HBO1 phosphorylation. (**A**) *DAB2IP^+/+^* and DAB2I*P^−/−^* MEFs cell lysates were immunoprecipitated with anti-HBO1 antibodies, and phosphorylation of HBO1 on CDK-target SP motifs (p-SP) was determined via immunoblotting. Right panel: Phosphorylation of HBO1 on its SP motifs was normalized to the total HBO1 protein level, then graphed for three independent experiments. Data are presented as mean ± s.d. from three independent experiments. The Wilcoxon rank-sum test was performed to assess statistical significance, **P < 0.01. (**B**) *DAB2IP* knockdown and control HeLa cell lysates were immunoprecipitated with anti-HBO1 antibodies. Phosphorylation of HBO1 was detected by immunoblotting using an antibody that recognizes CDK-target SP motifs (p-SP). Right panel: Phosphorylation of HBO1 on its SP motifs was normalized to the total HBO1 protein level, then graphed for three independent experiments. Data are presented as mean ± s.d. from four independent experiments. Wilcoxon rank-sum test was performed to assess statistical significance, **P < 0.01. (**C**) FLAG-tagged PLK1 was transiently expressed in *DAB2IP*-knockdown and control HeLa cells, FLAG-PLK1 was immunoprecipitated using anti-FLAG antibodies, and the ability of PLK1 to interact with DAB2IP and HBO1 was assessed via immunoblotting. Right panel: The amount of HBO1 that co-immunoprecipitated with PLK1 was calculated, normalized to the amount of FLAG-PLK1 that immunoprecipitated, and this was graphed by combining four independent experiments. Data are presented as mean ± s.d. from four independent experiments. The Wilcoxon rank-sum test was performed to assess statistical significance, **P < 0.01. (**D**) and (**E**) HeLa cells were transfected with siRNA against *DAB2IP* and a non-specific control for 48 h and were fixed for PLA staining (green) using anti-PLK1 and anti-HBO1 antibodies; anti-PLK1 only and anti-HBO1 only were adopted as negative controls. Representative images (**D**) of PLA foci in both groups. Scale bar = 10 μm. (**E**) The quantification of PLA spots per nucleus in DAB2IP-, HBO1-depletion, and control HeLa cells. *n* > 100 from three independent experiments. The horizontal bars represent the mean of each group. The Wilcoxon rank-sum test was used to examine statistical significance, ***P < 0.001.

In our previous study, we reported that DAB2IP interacts with PLK1 to regulate mitosis [11]. Beyond its role in mitosis, PLK1 is a crucial regulator of DNA replication and response to replication stress through its interaction with and phosphorylation of HBO1, facilitating the loading of the MCM complex onto chromatin. [22, 36–39]. These previous observations drove our hypothesis that DAB2IP regulates DNA replication by modulating the PLK1-HBO1 signaling axis. It was previously reported that CDK1 phosphorylates HBO1 at threonines 85 and 88, which are threonines followed by proline residues (pTP motif) [22]. These phosphorylated TP sites serve as docking platforms for the recruitment and activation of PLK1. Subsequently, activated PLK1 phosphorylates HBO1 at serine 57, also followed by a proline residue (pSP motif). This phosphorylation event is crucial for the loading of the MCM complex onto chromatin and the promotion of DNA replication [22]. To investigate the influence of DAB2IP on phosphorylation of HBO1, we immunoprecipitated HBO1 from *DAB2IP^−/−^* and *DAB2IP^+/+^* MEFs and observed a substantial reduction in HBO1 phosphorylation at SP sites in *DAB2IP^−/−^* MEFs compared to *DAB2IP^+/+^* MEFs (Fig. 5A). Additionally, we did not observe HBO1-specific pTP immunostaining (Supplementary Fig. S3A), indicating that DAB2IP influences PLK1-mediated, but not CDK1-mediated, phosphorylation of HBO1. Knockdown of DAB2IP expression in HeLa cells also resulted in a diminished phosphorylation of HBO1 at SP sites (Fig. 5B). Based on these findings, we hypothesized that DAB2IP may serve as a scaffolding protein, stabilizing the interaction between HBO1 and PLK1. To test this, we examined whether DAB2IP regulates the interaction between FLAG-tagged PLK1 and endogenous HBO1. Our results demonstrated a significant reduction in the interaction between PLK1 and HBO1 in *DAB2IP* knockdown cells (Fig. 5C). The PLA assay using antibodies against PLK1 and HBO1 also showed that *DAB2IP* depletion significantly impaired the formation of PLA foci in the nucleus (Fig. 5D and E). Additionally, we also found that the PLK1 inhibitor BI2536 significantly inhibited histone H3 K14 acetylation and MCM2 phosphorylation in both DAB2IP-expressing MEFs and HeLa cells (Supplementary Fig. S3B and C). In contrast, BI2536 only slightly blocked H3K14 acetylation and MCM2 phosphorylation in *DAB2IP*-deficient cells. Our findings indicate that DAB2IP interacts with both HBO1 and PLK1, playing a crucial role in stabilizing the PLK1-HBO1 interaction. This stabilization facilitates PLK1-mediated phosphorylation of HBO1, which in turn promotes HBO1-mediated signaling necessary for DNA replication initiation. By influencing these interactions and phosphorylation events, DAB2IP significantly contributes to the proper progression of DNA replication.

### ATR inhibition contributes to HBO1 phosphorylation by promoting CDK1-dependent DAB2IP-PLK1 association and subsequent activation of PLK1

**Figure 6.**
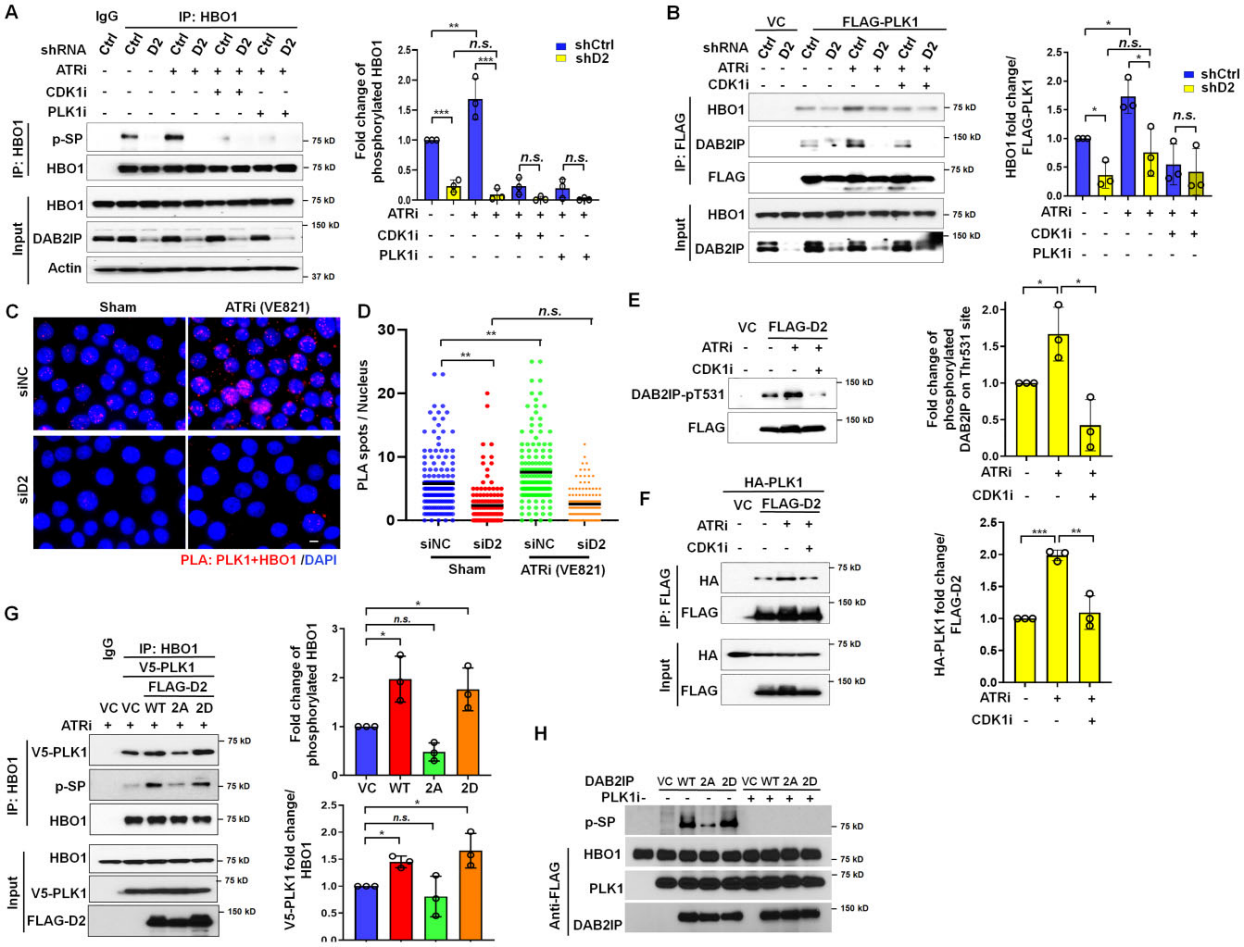
ATR inhibition promotes CDK1-mediated DAB2IP phosphorylation, which contributes to stable PLK1-HBO1 interaction and PLK1-mediated phosphorylation of HBO1. (**A**) *DAB2IP* knockdown and control HeLa cells were treated with vehicle, 5 µM VE-821, 5 µM VE-821 + 5 µM RO3306, or 5 µM VE-821 + 5 µM RO3306 + 10 nM BI2536, followed by immunoprecipitation of HBO1. Phosphorylation of HBO1 using phospho-SP antibodies was determined by immunoblotting. Right panel: The amount of phosphorylated HBO1 on SP sites was calculated, normalized to the amount of HBO1 that immunoprecipitated, and this was graphed by combining three independent experiments. Data are presented as mean ± s.d. from three independent experiments. One-way ANOVA test was performed to assess statistical significance, **P < 0.01, ***P < 0.001. (**B**) FLAG-tagged PLK1 was transiently expressed in DAB2IP knockdown and control HeLa cells. At 48 h later, cells were treated with vehicle, 5 µM VE-821, 5 µM VE-821 + 5 µM RO3306, or 5 µM VE-821 + 5 µM RO3306 + 10 nM BI2536, followed by immunoprecipitation of FLAG-PLK1 using anti-FLAG antibodies. The ability of HBO1 and DAB2IP to interact with PLK1 was assessed via immunoblotting. Right panel: The amount of HBO1 that co-immunoprecipitated with PLK1 was calculated, normalized to the amount of FLAG-PLK1 that immunoprecipitated, and this was graphed by combining three independent experiments. Data are presented as mean ± s.d. from three independent experiments. One-way ANOVA test was performed to assess statistical significance, *P < 0.05, **P < 0.01, ***P < 0.001. (**C**) and (**D**) *DAB2IP* knockdown and control HeLa cells were treated with vehicle and 5 µM VE-821, followed by PLA staining using anti-PLK1 and anti-HBO1 antibodies. Representative images (**C**) of PLA foci in indicated groups. Scale bar = 10 μm. (**D**) The quantification of PLA spots per nucleus in indicated groups of HeLa cells. *n* > 100 from three independent experiments. The horizontal bars represent the mean of each group. One-way ANOVA test was used to examine statistical significance, “*n.s*.” = no significance; **P < 0.01. (**E**) FLAG-tagged DAB2IP was transiently expressed in HeLa cells. 48 h later, cells were treated with vehicle, 5 µM VE-821, or 5 µM VE-821 + 5 µM RO3306, followed by immunoprecipitation of FLAG-DAB2IP using anti-FLAG antibodies. Immunoblotting was performed to assess total phosphorylation of DAB2IP at phospho-TP sites (pTP) and at phospho-T531 (DAB2IP-pT531). Right panel: The amount of phosphorylated DAB2IP on its Thr531 site was calculated, normalized to the amount of FLAG-DAB2IP that immunoprecipitated, and this was graphed by combining three independent experiments. Data are presented as mean ± s.d. from three independent experiments. One-way ANOVA test was performed to assess statistical significance, *P < 0.05. (**F**) FLAG-tagged DAB2IP and HA-tagged PLK1 were transiently expressed in HeLa cells. 48 h later, cells were treated with vehicle, 5 µM VE-821, or 5 µM VE-821 + 5 µM RO3306, followed by immunoprecipitation of FLAG-DAB2IP using anti-FLAG antibodies. The ability of FLAG-DAB2IP and HA-PLK1 was determined by immunoblotting. Right panel: The amount of HA-PLK1 that co-immunoprecipitated with FLAG-DAB2IP was calculated, normalized to the amount of FLAG-DAB2IP that immunoprecipitated, and this was graphed by combining three independent experiments. Data are presented as mean ± s.d. from three independent experiments. One-way ANOVA test was performed to assess statistical significance, **P < 0.01, ***P < 0.001. (**G**) shRNA-resistant DAB2IP (rWT), shRNA-resistant DAB2IP 2A (r2A), and shRNA-resistant DAB2IP 2D (r2D) constructs were co-transfected with V5-tagged PLK1 in shRNA-mediated stable DAB2IP suppressing HeLa cells. 48 h later, the cells were treated with 5 µM VE-821, followed by HBO1 immunoprecipitation. The ability of PLK1 to interact with HBO1 and phosphorylation of HBO1 on pSP sites was assessed via immunoblotting. The intensity of phosphorylated HBO1 on pSP sites (upper panel) and the interaction with V5-PLK1 (lower panel) were normalized to FLAG-vector control (VC), and V5-PLK1 co-transfected cells. Data are presented as mean ± s.d. from three independent experiments. One-way ANOVA test was performed to assess statistical significance, *P < 0.05, and “*n.s*.” = no significance. (**H**) DAB2IP significantly enhances PLK1-mediated phosphorylation of HBO1 on its SP sites *in vitro. In vitro* kinase assays were performed using purified FLAG-tagged PLK1 and FLAG-tagged HBO1, with or without PLK1 inhibitor treatment, in the presence of purified FLAG-tagged DAB2IP, DAB2IP-2A, or DAB2IP-2D. Phosphorylation of HBO1 was assessed via immunoblotting using anti-pSP motif antibodies.

Recent studies have demonstrated the crucial involvement of the ATR signaling pathway in restricting CDK1 activation and replication origin firing during normal DNA replication [40, 41]. To explore the impact of ATR on PLK1 activity toward HBO1 and the role of DAB2IP in this process, we treated *DAB2IP*-depleted and control HeLa cells with various combinations of inhibitors, including VE-821 (ATR inhibitor), VE-821 + RO3306 (combination of ATR and CDK1 inhibitor), and VE-821 + BI2536 (combination of ATR and PLK1 inhibitor). HBO1 was immunoprecipitated from treated cells, and the phosphorylation of HBO1 at SP sites was assessed. The data revealed that the ATR inhibitor notably enhanced the phosphorylation of HBO1 on SP sites. However, the addition of CDK1 or PLK1 inhibitors completely abolished the SP phosphorylation of HBO1, even in response to ATR inhibition (Fig. 6A). Furthermore, in *DAB2IP*-depleted cells, ATR inhibition failed to enhance the SP phosphorylation of HBO1 (Fig. 6A). This finding suggests that DAB2IP plays an essential role in facilitating CDK1-primed and PLK1-mediated HBO1 phosphorylation. Additionally, we observed that ATR promoted the interaction between PLK1 and HBO1 in control HeLa cells, whereas its impact was only marginal in DAB2IP-depleted HeLa cells (Fig. 6B). PLA assay also showed that the ATR inhibitor significantly enhanced the association between PLK1 and HBO1 in control HeLa cells, but not in *DAB2IP*-knocked-down HeLa cells (Fig. 6C and D).

In our previous study, we demonstrated that CDK1 phosphorylates DAB2IP at Thr-531 and Thr-546. Therefore, we investigated if CDK1-mediated phosphorylation of DAB2IP primes PLK1 and activates its activity toward HBO1 [11]. To assess this, FLAG-tagged wild-type (WT) DAB2IP and the DAB2IP-Thr531Ala/Thr-546Ala double site mutants (DAB2IP-2A) were expressed in HeLa cells. We then synchronized HeLa cells at the G1/S boundary using a double thymidine blockage. After release from the block, we immunoprecipitated the FLAG-tagged DAB2IP from cells at various cell cycle phases, including G1/S, S, and mitosis. Our findings revealed that DAB2IP, but not the DAB2IP-2A mutant, is phosphorylated on TP sites in each phase of the cell cycle (Supplementary Fig. S4A). Using a phospho-Thr531 antibody, we observed that treatment with ATRi increased phosphorylation at T531, which was attenuated with co-treatment with CDK1i (Fig. 6E). Next, we examined if modulation of DAB2IP phosphorylation by ATR and/or CDK1 affects the interaction between DAB2IP and PLK1 as well as the activation of PLK1. We found that pretreatment with the ATRi significantly increased the interaction between PLK1 and DAB2IP (Fig. 6B and F). Conversely, co-treatment of CDK1i and ATRi eliminated the ATR inhibitor-mediated enhancement of DAB2IP-PLK1 association (Fig. 6B and F). We then determined if DAB2IP phosphorylation affects HBO1 phosphorylation and the HBO1-PLK1 interaction. Transient expression of WT and DAB2IP phosphorylation mimic mutants (DAB2IP-2D) rescued HBO1 phosphorylation and promoted increased interaction between HBO1 and PLK1, which was not observed in DAB2IP knockout cells that were rescued with the DAB2IP 2A mutant (Fig. 6G). The mutation of DAB2IP at Thr-531 and Thr-546 does not influence the interaction between DAB2IP and HBO1 (Supplementary Fig. S4B). To validate the role of phosphorylated DAB2IP in PLK1-mediated HBO1 phosphorylation, we purified FLAG-tagged WT DAB2IP, DAB2IP-2A, and DAB2IP-2D, as well as FLAG-tagged PLK1 and FLAG-HBO1, from HeLa cells treated with ATRi (Supplementary Fig. S4C and D). FLAG-tagged PLK1 and HBO1 proteins were treated with λPP to remove endogenous phosphorylation signals and then mixed together to perform *in vitro* kinase assays. We found that WT FLAG-tagged DAB2IP and DAB2IP-2D proteins, but not the DAB2IP-2A mutant, significantly enhanced PLK1-mediated phosphorylation of HBO1 on its SP sites (Fig. 6H). Together, these data indicate that CDK1-mediated phosphorylation of DAB2IP generates a docking site for PLK1, facilitating DAB2IP-PLK1 interaction and promoting PLK1-mediated phosphorylation of HBO1.

### Phosphorylation of DAB2IP is essential for maintaining genomic stability

**Figure 7.**
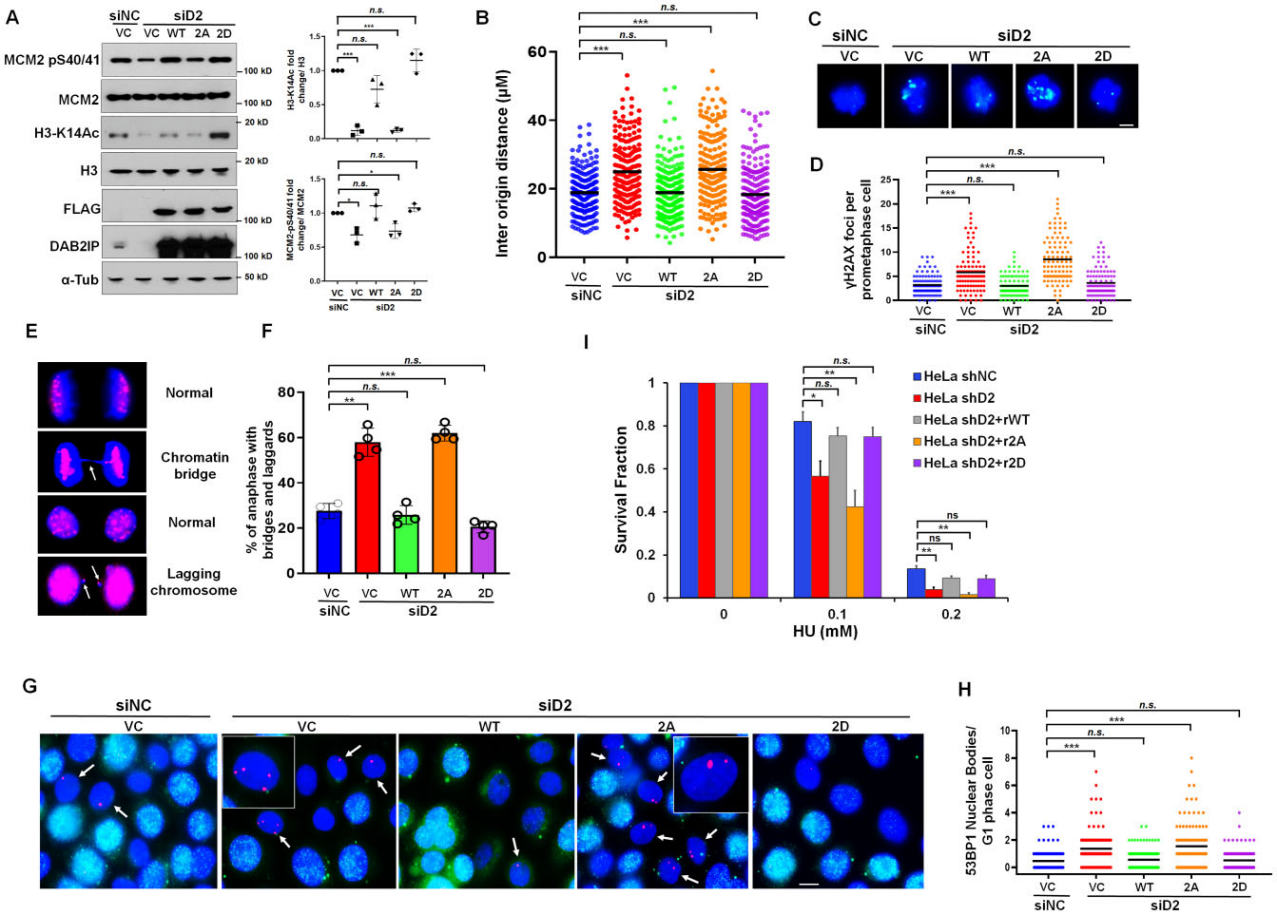
Phosphorylation of DAB2IP protects cells against replication stress by suppressing the accumulation of incomplete DNA replication. (**A**) Immunoblotting analysis assessing MCM2-pS4-/41, MCM2, H3K14 acetylation, H3, and DAB2IP in HeLa cells with siRNA-mediated *DAB2IP* suppression and overexpression of siRNA-resistant DAB2IP (rWT), siRNA-resistant DAB2IP 2A (r2A), and siRNA-resistant DAB2IP 2D (r2D) constructs. The intensity of acetylated histone H3 on its K14 site (upper panel) and phosphorylated MCM2-S40/41 (lower panel) was normalized to total histone H3 and MCM2 proteins, respectively. Data are presented as mean ± s.d. from three independent experiments. One-way ANOVA test was performed to assess statistical significance, *P < 0.05, ***P < 0.001, and “*n.s*.” = no significance. (**B**) HeLa cells were transfected with DAB2IP-targeted siRNA and overexpressed with siRNA-resistant DAB2IP (rWT), siRNA-resistant DAB2IP 2A (r2A), and siRNA-resistant DAB2IP2D (r2D) constructs. DNA fiber analysis was performed in the indicated groups of HeLa cells after sequential labeling with IdU (10 min) and CldU (20 min). The inter-origin distances are quantified. n > 200 from three independent experiments. The horizontal bars represent the mean of each group. One-way ANOVA test was used to examine statistical significance, “*n.s*.” = no significance; ***P < 0.001. (**C**) and (**D**) HeLa cells were transfected with siRNA to suppress *DAB2IP* expression or were co-transfected with siRNA and siRNA-resistant DAB2IP (rWT), siRNA-resistant DAB2IP 2A (r2A), and siRNA-resistant DAB2IP 2D (r2D) constructs. Cells were treated with 2 mM HU for 2 h. At 24 h after HU treatment, immunofluorescent staining was performed using anti-γH2AX (green) antibody. (**C**) Representative images of γH2AX foci in prometaphase cells. (**D**) The γH2AX foci number per cell in prometaphase was assessed in different groups (*n* = 100/group from three independent experiments). The horizontal bars represent the mean of each group. One-way ANOVA test was used to examine statistical significance, ***P < 0.001; “*n.s*.” = no significance. Scale bar = 10 μm. (**E**) and (**F**) Immunofluorescent staining using anti-Crest (red) and anti-α-tubulin (green) antibodies was performed after the treatment described in panel (**B**). Representative images of anaphase cells with chromatin bridges and lagging chromosomes are shown in (**E**). (**F**) The percentage of anaphase cells with chromatin bridges and lagging chromosomes was assessed in different groups (Data are presented as mean ± s.d. from three independent experiments; At least 200 anaphase cells were counted in each group. One-way ANOVA was performed to assess statistical significance, **P < 0.01; ***P < 0.001; and “*n.s*.” = no significance. Scale bar = 10 μm. (**G**) and (**H**) Immunofluorescent staining using anti-53BP1 (red) and –cyclin A (green) antibodies was performed after the treatment described in panel (**B**). Representative images of 53BP1 nuclear bodies in the G1 cell-cycle phase (53BP1-nuclear bodies in cyclin A-negative cells) are shown in (**G**). (**H**) The 53BP1 nuclear foci number per G1 cell was assessed in different groups (*n* = 100/group from three independent experiments. The horizontal bars represent the mean of each group. One-way ANOVA test was used to examine statistical significance, ***P < 0.001; “*n.s*.” = no significance.. Scale bar = 10 μm. (**I**) HeLa cells were transfected with siRNA to suppress *DAB2IP* expression or were co-transfected with siRNA and siRNA-resistant DAB2IP (rWT), siRNA-resistant DAB2IP 2A (r2A), and siRNA-resistant DAB2IP 2D (r2D) constructs. Cells were analyzed for their colony-forming ability following treatment with HU. Data are presented as mean ± s.d. from three independent experiments. One-way ANOVA test was performed to assess statistical significance, *P < 0.05; **P < 0.01; and “*n.s*.” = no significance.

To further gain insight into the role of phosphorylated DAB2IP in the regulation of DNA replication and genome maintenance, we reintroduced siRNA-resistant wild-type DAB2IP, DAB2IP-2A, and DAB2IP-2D into DAB2IP-depleted HeLa cells. The expression of WT DAB2IP and the DAB2IP-2D mutant, but not DAB2IP-2A, restored acetylation of H3 on K14 and the phosphorylation of MCM2 on S40/41, indicating that phosphorylation of DAB2IP promotes HBO1-directed activities (Fig. 7A). We also found that the inter-origin distance in DAB2IP-depleted cells was significantly restored by both the WT and the phosphorylation-mimic mutant of DAB2IP, but not by its non-phosphorylatable form (Fig. 7B). Dysregulated DNA replication rate can cause replication stress, leading to accumulation of DNA damage during mitosis, and ultimately resulting in the formation of ultrafine chromatin bridges (UFBs) during anaphase [42, 43]. We postulated that replication stress associated with DAB2IP loss would lead to these events. Consistent with this hypothesis, loss of DAB2IP resulted in a marked increase in the number of mitotic DSBs as assessed by γH2AX foci per prometaphase cell (Fig. 7C and D). Moreover, this correlated with increased formation of anaphase bridges and lagging chromosomes, and subsequent formation of 53BP1 bodies in G1 phase of the cell cycle (Fig. 7E–H). In contrast, the heightened genomic instability observed upon loss of DAB2IP was effectively reversed by expressing wild-type DAB2IP and DAB2IP-2D proteins (Fig. 7C–H). However, the expression of the siRNA-resistant DAB2IP-2A mutant did not exhibit the same rescue effect. Additionally, our findings indicate that phosphorylated DAB2IP plays a crucial role in maintaining resistance to HU treatment (Fig. 7I). These results demonstrate that phosphorylation of DAB2IP at Thr531 and Thr546 is necessary for proper DNA replication and the maintenance of genomic stability. Collectively, our study indicates that the (ATR-) CDK1-DAB2IP-PLK1-HBO1 axis regulates DNA replication by promoting HBO1-mediated acetylation of histone H3, leading to MCM loading and onset of DNA replication, and if this process is dysregulated, it leads to DNA replication stress and genomic instability (Supplementary Fig. S5). We postulate that this process is vital for homeostasis in normal cycling cells and in response to cellular stress.

## Discussion

In this study, we uncovered a previously unknown function of DAB2IP in regulating the initiation of DNA replication. Previously, we established that DAB2IP is a novel regulator of PLK1 in the context of mitotic progression [10, 11]. PLK1 is a critical kinase involved in orchestrating various aspects of mitotic progression, and its loss or inactivation leads to severe mitotic aberrations and cell death. Additionally, PLK1 plays a role in DNA replication progression, both in cycling cells and in response to stress exposure [22, 37]. It has been shown to directly interact with members of the MCM complex, including MCM2, MCM3, and MCM7 [20]. Depletion of PLK1 disrupts the proper chromatin loading of the MCM complex and reduces DNA synthesis during the S phase of the first cell cycle following depletion [38]. This is supported by another study demonstrating that PLK1 phosphorylates Ser-57 of HBO1, which is crucial for MCM complex assembly [22]. The polo-box domain (PBD) of PLK1 has a propensity to bind to proteins that are primed-phosphorylated by CDKs, typically represented by the consensus sequence S-pS/pT-P/X [44]. The binding of the PBD domain to these primed-phosphorylated proteins triggers the release of PLK1’s *N*-terminus kinase domain, leading to its activation. CDK1-mediated priming phosphorylation of HBO1 plays a crucial role in facilitating the interaction between PLK1 and HBO1 [22]. This priming phosphorylation event by CDK1 primes HBO1 for subsequent phosphorylation by PLK1, further regulating HBO1’s functions towards MCM chromatin loading. Our data provide evidence for the interaction of DAB2IP with both HBO1 and PLK1 through adjacent motifs (Fig. 3). This indicates a novel role for DAB2IP as a scaffolding protein that can simultaneously recruit HBO1 and PLK1, leading to the specific activation of PLK1 on its target HBO1 (Fig. 5). Supporting this concept is the observation that although the phosphorylation-deficient mutant of DAB2IP (Thr531Ala and Thr546Ala) does not affect the interaction between DAB2IP and HBO1, it significantly impairs the effect of DAB2IP on the association between PLK1 and HBO1 as well as the PLK1-mediated phosphorylation of HBO1 (Fig. 6).

The ATR-Chk1 signaling pathway plays a crucial role in the cell cycle checkpoint response to genotoxic stress by inhibiting CDK activity, allowing cells sufficient time to resolve the stress and maintain genomic integrity. Under normal conditions, ATR is activated at relatively low levels to regulate the S/G2 cell cycle transition, ensuring it proceeds only after DNA replication is complete [45]. The ATR-Chk1 signaling pathway inhibits CDK1-mediated phosphorylation of Rif1 on Ser2205 in mammalian cells [41]. Rif1 interacts with protein phosphatase 1 (PP1), recruiting it to chromatin to counteract the phosphorylation of MCM proteins induced by CDC7 and CDK2, thus regulating the initiation of DNA replication [46]. Inhibition of ATR-Chk1 signaling increases CDK1-mediated phosphorylation of Rif1, disrupting the interaction between Rif1 and PP1 and resulting in the activation of replication origin firing. A study using *Xenopus* as a model system revealed that PLK1 phosphorylates the same conserved site of Rif1, disrupting the Rif1-PP1 interaction [39]. Although it remains unknown whether ATR directly suppresses the activity of PLK1 on Rif1, our study provides evidence that ATR regulates PLK1-mediated phosphorylation of HBO1. Furthermore, our findings suggest that this regulation by ATR relies on CDK1-primed phosphorylation. Direct evidence comes from the observation that the inhibition of ATR enhances HBO1 phosphorylation, which can be suppressed by PLK1 and CDK1 inhibitors. Moreover, our study unveils the role of DAB2IP in supporting CDK1 and PLK1 activation on their targets after ATR inhibition. In the context of DAB2IP depletion, the ATR inhibitor only slightly enhances the interaction between HBO1 and PLK1 as well as the PLK1-mediated phosphorylation of HBO1 (Fig. 6). Further investigation is required to fully elucidate the specific contribution of DAB2IP in ATR-suppression-related molecular events during replication regulation, such as the dissociation of Rif1-PP1 and the activation of the MCM complex. Our data showing that DAB2IP promotes MCM2 phosphorylation in both *in vivo* and *in vitro* provide support for this hypothesis (Fig. 4). In addition to the important function in replication origin firing, MCM2 Ser41 phosphorylation also contributes to DNA replication fork progression [47]. Our DNA fiber data show that DAB2IP-deficient MEFs and C4-2 cells exhibited increased inter-replication origin distance and decreased DNA replication tracks (Fig. 1 and Supplementary Fig. S1). It is worth noting that DAB2IP-knockdown HeLa cells exhibit increased replication fork speed compared to control cells. A recent study revealed the existence of two independent DNA replication patterns in a *Xenopus* model system, referred to as fast and slow replication modes, characterized by opposite origin firing rates [48]. PLK1 plays an essential role in regulating this spatial replication program. Loss of PLK1 abolishes the spatiotemporal exclusivity of these two processes as it results in increased fork speed but decreased origin firing in the fast-cycling model system with a short S phase. We postulate that the increased replication fork speed observed in HeLa cells following DAB2IP knockdown may be due to a distinct replication mode that is different from those observed in the *DAB2IP^−/−^* MEFs and C4-2 cells. Further work is required to fully elucidate the function of DAB2IP in the replication fork.

PLK1 is widely recognized for its oncogenic properties, given its critical role in driving cell cycle progression in tumor cells. However, emerging evidence suggests that PLK1 can also function as a tumor suppressor in specific contexts. For instance, overexpression of PLK1 can lead to abnormal chromosome segregation and cytokinesis failure, generating polyploid cells with reduced proliferative activity [49]. *In vivo* studies have demonstrated that PLK1-overexpression delays Kras- and Her2-induced mammary gland tumor development [49]. Conversely, inhibition of PLK1 increased intestinal tumors in two independent *Apc^Min/+^* mouse models [50]. These findings suggest a complex role for PLK1 in tumor biology, where it not only promotes cell cycle progression but also suppresses genomic instability—a major driving force of tumor development. PLK1 promotes genomic stability by ensuring proper mitosis progression and preventing incomplete DNA synthesis and loss of DNA integrity. [38]. In our study, we observed the accumulation of DNA damage and subsequent chromosomal abnormalities in *DAB2IP^−/−^* cells, particularly under stress exposure (Fig. 2). This suggests that the suppression of genome instability via the PLK1-mediated signaling pathway is likely a crucial aspect of DAB2IP’s ability to inhibit tumor development. Prolonged DNA synthesis leads to incomplete replication of genomic DNA, especially at the difficult-to-replicate regions such as common fragile sites (CFS). This incomplete replication results in the accumulation of mitotic ultrafine bridges (UFBs) [51]. The resolution of UFBs is commonly accompanied by the generation of micronuclei and G1-phase 53BP1 nuclear bodies, providing fuel for the selection of cancer evolution. Our data show increased levels of UFBs during anaphase and G1-phase 53BP1 nuclear bodies in *DAB2IP*-deficient or non-phosphorylatable DAB2IP cells in response to replication stress. These observations suggest that DAB2IP plays a crucial role in maintaining genomic stability and suppressing tumor development, at least in part through a PLK1-dependent pathway (Fig. 7).

We propose the following model for DAB2IP’s role in DNA replication initiation regulation (Supplementary Fig. S5). DAB2IP interacts with HBO1 in the nucleus through its GAP domain. CDK1-mediated phosphorylation of DAB2IP facilitates the recruitment of PLK1, acting as a scaffold to bridge HBO1 and PLK1 and subsequently activating PLK1’s kinase activity toward HBO1. The ATR-Chk1 signaling pathway modulates the activity of the CDK1-DAB2IP-PLK1-HBO1 axis, thereby limiting replication origin activity to ensure proper timing and coordination of DNA replication. Loss of DAB2IP or its phosphorylation by CDK1 leads to prolonged replication progression and incomplete global DNA replication. This disruption results in DNA damage and genomic instability, contributing to potential tumor development. This model underscores the pivotal role of DAB2IP in maintaining genomic stability by regulating the initiation and progression of DNA replication through its interactions with HBO1 and PLK1, under the control of ATR signaling. The precise orchestration of these interactions ensures the fidelity of DNA replication and the prevention of genomic instability.

## Supplementary Material

gkaf1179_Supplemental_Files

## Data Availability

All data are provided in the supplementary data files.
