# Supplementary material for: Nuclear DAB2IP regulates DNA replication initiation through activating PLK1-mediated HBO1 phosphorylation
Source: Nucleic Acids Res. 2025 Nov 20;53(21):gkaf1179. doi: 10.1093/nar/gkaf1179 (PMC12630137; doi:10.1093/nar/gkaf1179)
Supplement: gkaf1179_Supplemental_Files [file gkaf1179_supplemental_files.zip › Supplemental Information clean R2.pdf]

Supplementary Information

Nuclear DAB2IP regulates DNA replication initiation through activating PLK1-mediated HBO1 phosphorylation

Zeng-Fu Shang[1*†], Lan Yu[2†], Ciara Newman[1], Wei-Min Chen[1], Grant W. Birdsong[1], Brett C. Sharp[1], Michael D. Story[1,4], Debabrata Saha[1,3*], and Anthony J. Davis[1,3*]

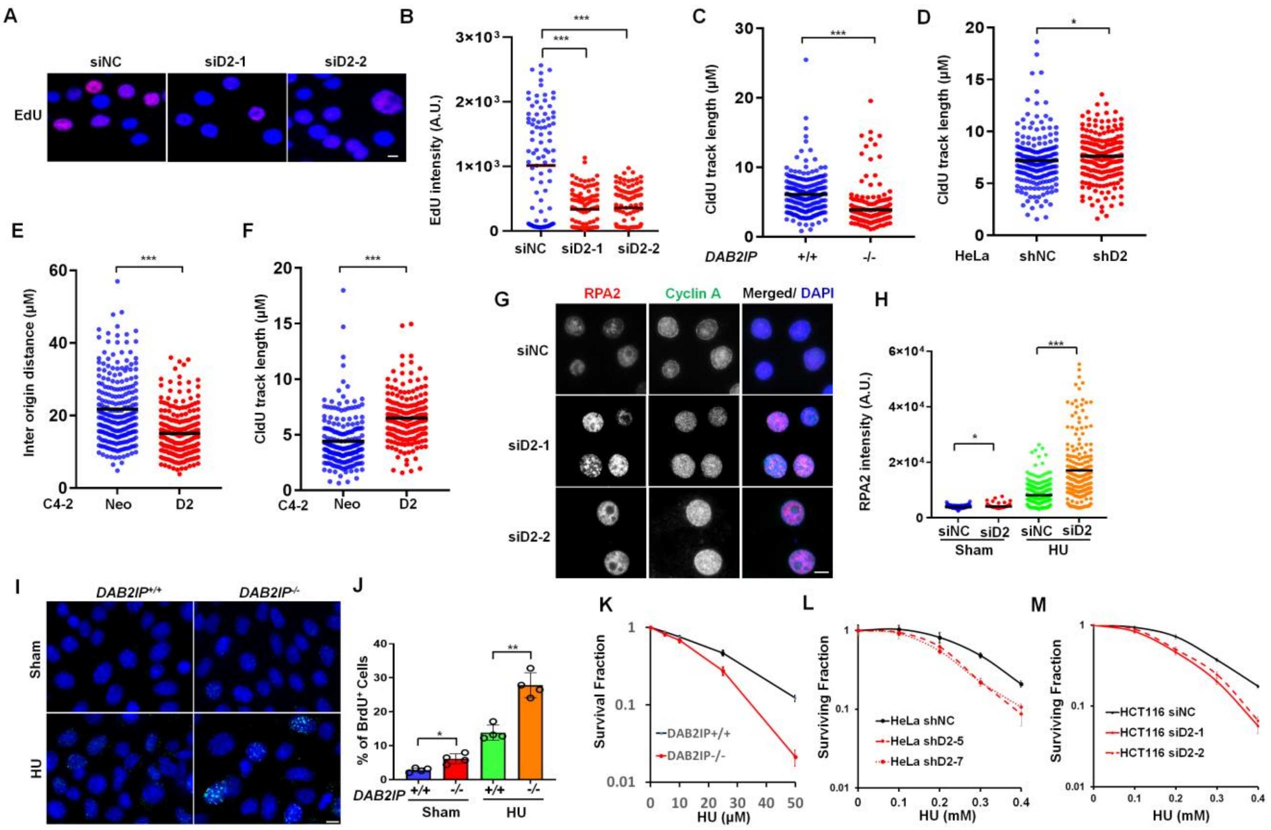

Figure S1. DAB2IP loss decreases DNA replication initiation and induces replication stress. (**A**) and (**B**) *DAB2IP* knockdown and control HeLa cells were pulse-labeled with 50 μM EdU for 30 min. Data are presented as EdU densities in nuclei (n > 100/condition). The horizontal bars represent the mean of each group. One-way ANOVA was used to examine statistical significance, $^{***}P < 0.001$. Scale bar = 10 μm. (**C**) and (**D**) DNA fibers in *DAB2IP*$^{+/+}$, *DAB2IP*$^{-/-}$ MEFs (**C**), *DAB2IP*-knocked down and control (shNC) HeLa cells (**D**) after sequential labeling with iododeoxyuridine (IdU, 10 min) and chlorodeoxyuridine (CldU, 20 min). CIdU tracks length are quantified. n > 200 from three independent experiments. The horizontal bars represent the mean of each group. Wilcoxon rank-sum test was used to examine statistical significance, $^{***}P < 0.001$. (**E**) and (**F**) DNA fibers in C4-2 Neo and C4-2 D2 cells after sequential labeling with ododeoxyuridine (IdU, 10 min) and chlorodeoxyuridine (CldU, 20 min). Scale bar = 10 μm. The inter-origin distance (**E**) and CIdU tracks length (**F**) of C4-2 Neo and D2 cells are quantified. n > 200 from three independent experiments. The horizontal bars represent the mean of each group. Wilcoxon rank-sum test was used to examine statistical significance, $^{***}P < 0.001$. (**G**) and (**H**) HeLa cells transfected with the siRNA against control and *DAB2IP*, and cells were treated with HU (2 mM) for 2 h, pre-extracted with 0.1% TX-100, and stained against anti-RPA2 and -Cyclin A antibodies. Data are presented as RPA2 intensity in Cyclin A^{+} nuclei (n > 100/condition). The horizontal bars represent the mean of each group. One-way ANOVA test was used to examine statistical significance, $^{**}P < 0.01$; $^{***}P < 0.001$. Scale bar = 10 μm. (**I**) and (**J**) *DAB2IP*$^{+/+}$ and *DAB2IP*$^{-/-}$ MEFs were labeled with BrdU (10 μM) for 30 h and then treated with HU (0.5 mM) for 2 h. BrdU-labeled single-stranded DNA was stained using an anti-BrdU antibody under non-denaturing condition. Data are presented mean ± s.d. from 4 independent experiments. One-way ANOVA test was performed to assess statistical significance, $^{*}P < 0.05$; $^{**}P < 0.01$. (**K**-**M**) Loss of DAB2IP results in increased sensitivity to HU. (**K**) *DAB2IP*$^{+/+}$ and DAB2IP$^{-/-}$ MEFs, *DAB2IP* knockdown and control HeLa cells (**L**) and HCT116 cells (**M**) were treated with the indicated doses of HU and plated for analysis of survival and colony-forming ability. Data are presented as mean ± s.d. from 3 independent experiments. Student's t-test (two-sided) was performed to assess statistical significance, $^{**}P < 0.01$; $^{***}P < 0.001$.

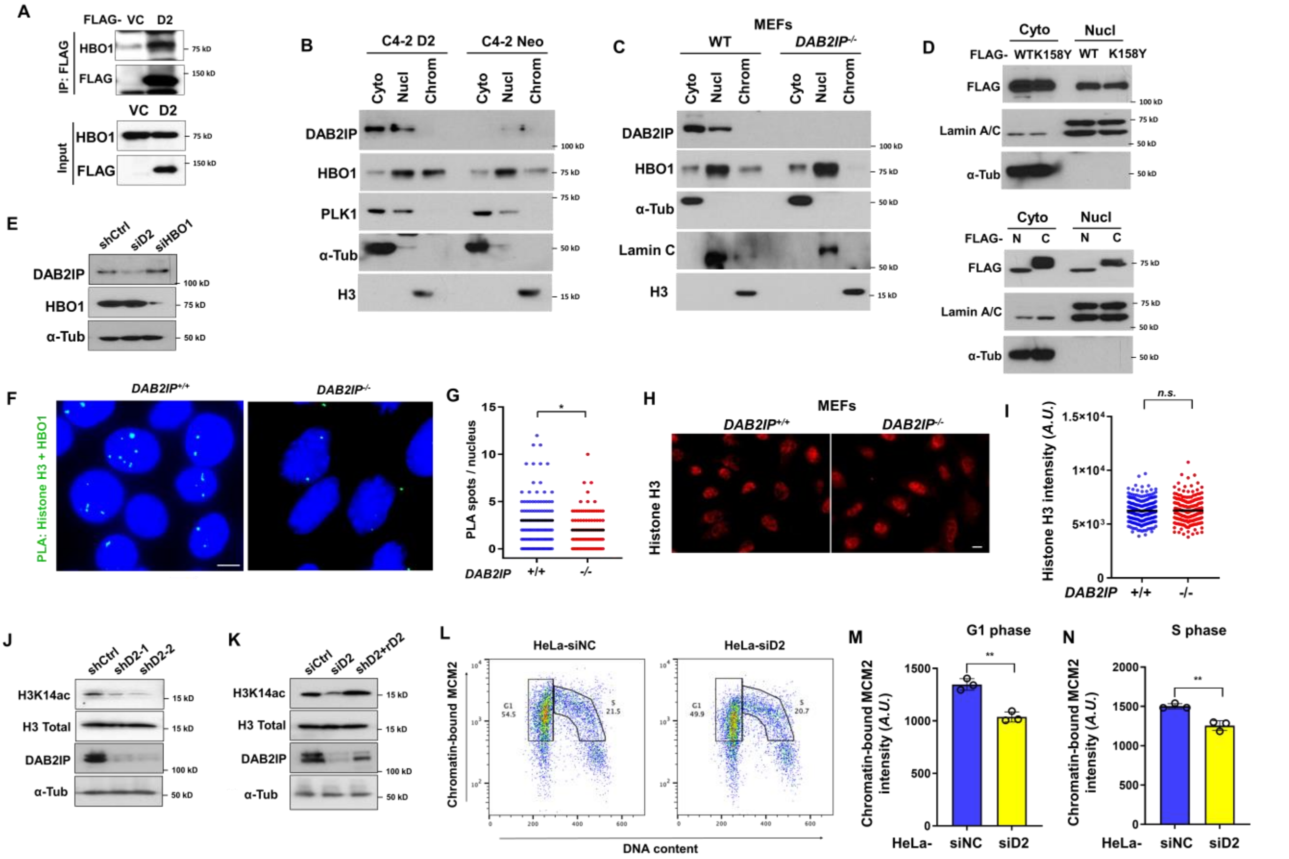

Figure S2. DAB2IP interacts with HBO1, promotes nuclear translocation of HBO1 and HBO1-mediated H3K14 acetylation. (**A**) FLAG-tagged DAB2IP was transiently expressed in HeLa cells and was subsequently immunoprecipitated using anti-FLAG antibodies. The ability of endogenous HBO1 to co-immunoprecipitate with FLAG-DAB2IP was assessed via immunoblotting. (**B**) and (**C**) Cytosol (Cyto), soluble nuclear (Nucl) and chromatin-enriched (Chrom) fractions were isolated from C4-2 Neo and C4-2 D2 cells (**B**), *DAB2IP*$^{+/+}$ and *DAB2IP*$^{-/-}$ MEFs (**C**) for immunoblotting to assess the sub-cellular localization of indicated proteins. (**D**) Plasmids expressing FLAG-tagged wild-type DAB2IP, DAB2IP-K158Y mutants, N-terminus and C-terminus truncations of DAB2IP were transfected into HeLa cells for 48 hours. Cells were harvested to isolate the cytosol and nuclear fractions for immunoblotting analysis using anti-FLAG and other indicated antibodies. (**E**) The HeLa cells were transfected with siRNA against *DAB2IP*, *HBO1*, and non-specific control. The knockdown efficiency was determined by immunoblotting. (**F**) and (**G**) *DAB2IP*$^{+/+}$ and *DAB2IP*$^{-/-}$ MEFs were fixed for PLA staining (green) using anti-histone H3 and anti-HBO1 antibodies. Representative images (**F**) of PLA foci in different groups. Scale bar= 10 μm. (**G**) The quantification of PLA spots per nucleus in DAB2IP-, HBO1-depletion and control HeLa cells. n > 100 from 3 independent experiments. The horizontal bars represent the mean of each group. Wilcoxon rank-sum test was used to examine statistical significance, $^{***}P < 0.001$. (**H**) Immunofluorescent staining of histone H3 in *DAB2IP*$^{+/+}$ and DAB2I*P*$^{-/-}$ MEFs. (**I**) Quantification of H3K14Ac intensities was performed (n > 100 from 3 independent experiments). The horizontal bars represent the mean of each group. Wilcoxon rank-sum test was used to examine statistical significance, $^{***}P < 0.001$. Scale bar = 10 μm. (**J**) Immunoblot analysis of H3K14 acetylation (H3K14ac), total H3, and the loading control alpha-tubulin (α-Tub) in *DAB2IP*-knockdown and control HeLa cells. (**K**) Immunoblot analysis of H3K14 acetylation (H3K14ac), total H3, and the loading control alpha-tubulin (α-Tub) in HeLa *DAB2IP*-knockdown, control, and *DAB2IP*-knockdown cells rescued via transient expression of DAB2IP (rD2). (**L**) Chromatin flow cytometry analysis of chromatin-bound MCM2 in *DAB2IP*-depleted and control HeLa cells. (**M**) and (**N**) Quantification of chromatin-bound MCM2 intensities in G1 (M) and S (N) phase were presented from 3 independent experiments. Wilcoxon rank-sum test was used to examine statistical significance, $^{**}P < 0.01$.

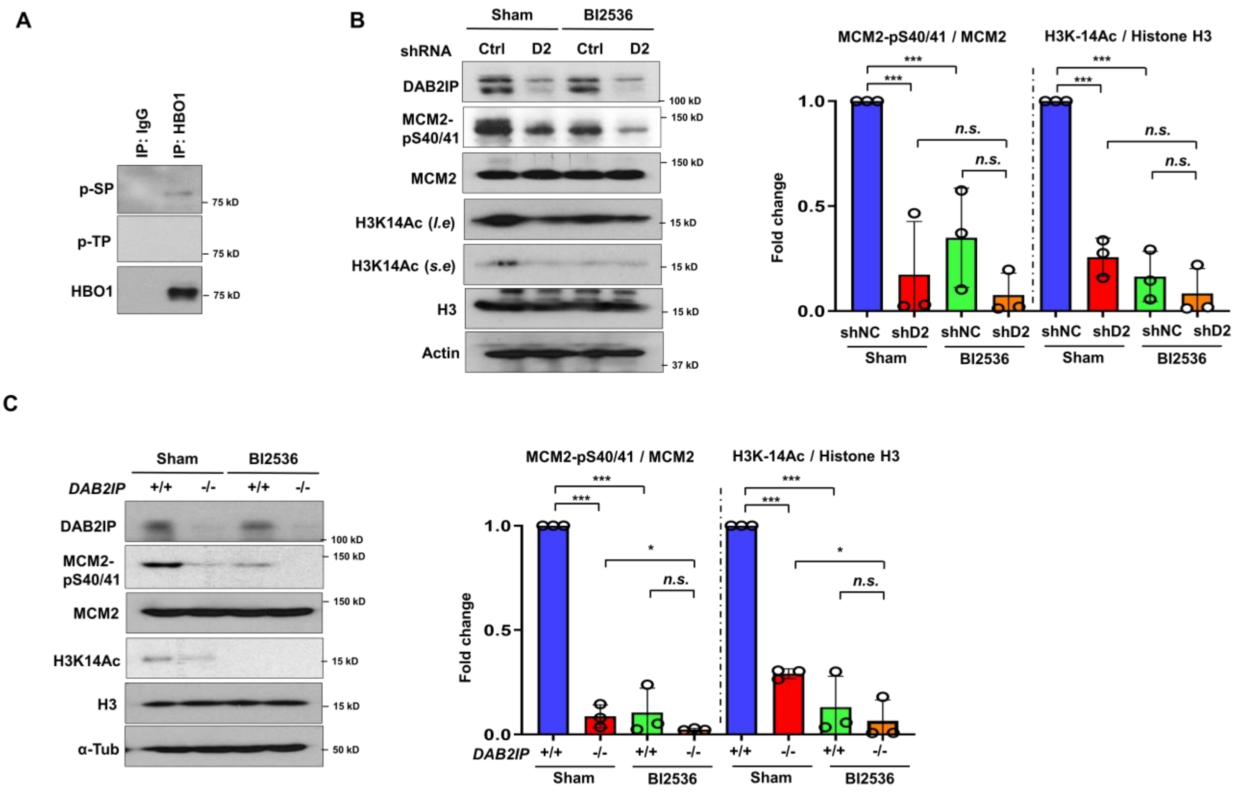

Figure S3. DAB2IP regulates PLK1-HBO1 pathway. (**A**) Endogenous HBO1 was immunoprecipitated from HeLa cells lysates and phosphorylation of HBO1 were assessed by immunoblotting using antibodies that recognizes CDK-targeted SP and TP motifs (p-SP and p-TP). (**B**) and (**C**) *DAB2IP*-knockdown and control HeLa cells (**B**), and *DAB2IP*$^{+/+}$ and *DAB2IP*$^{-/-}$ MEFs were incubated with or without 10 nM of PLK1 inhibitor (BI2536) for 4 hrs. Cells were harvested for immunoblotting analysis of the indicated proteins. Right panel: The levels of pMCM2 was normalized to the total MCM2 protein. The levels of H3K14Ac was normalized to the total histone H3 protein level, then graphed for three independent experiments. Data are presented as mean ± s.d. from 3 independent experiments. One-way ANOVA test was performed to assess statistical significance, $^{*}P < 0.05$, $^{**}P < 0.01$.

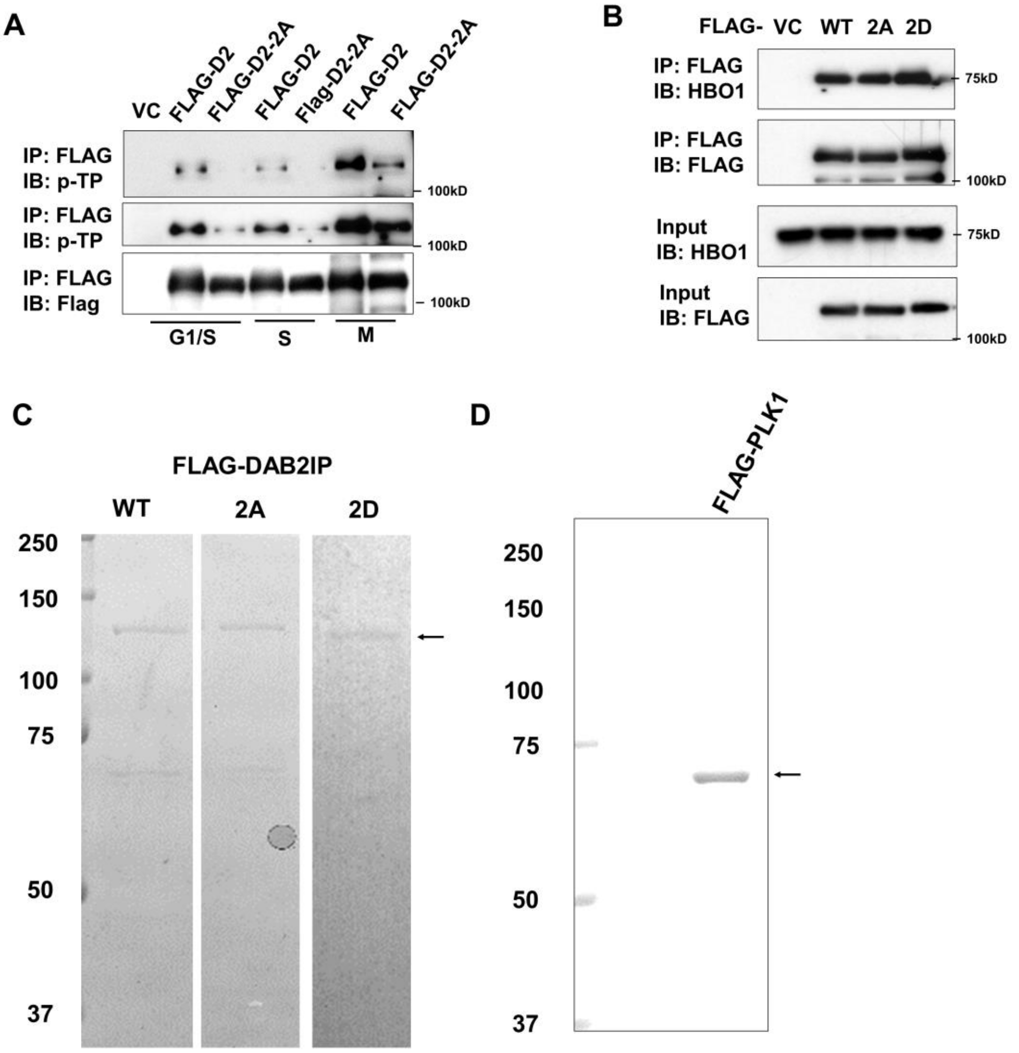

Figure S4. A. FLAG-tagged wild-type DAB2IP (D2) and DAB2IP Thr-531A/Thr-546A (D2-2A) double phosphorylation site mutant were transiently expressed in HeLa cells, the cells were then synchronized in G1 phase of the cell cycle via double thymidine block (DTB), and were subsequently released in fresh medium for 4 and 9 h to enrich S phase and G2/M phase cells, respectively. The D2 and D2-2A proteins were immunoprecipitated using anti-FLAG antibodies and phosphorylation of TP sites were assessed by immunoblotting. **B.** FLAG-tagged DAB2IP (WT), DAB2IP T531A/T546A (2A), DAB2IP T531D/T546D (2D), and empty vector (VD) were transiently expressed in HeLa cells, the proteins were immunoprecipitated using anti-FLAG antibodies, and the ability of HBO1 to co-immunoprecipitate with DAB2IP was assessed via immunoblotting. Coomassie blue staining gel of recombinant FLAG-tagged DAB2IP (WT), 2A and 2D **(C)** and FLAG-tagged PLK1 **(D)**.

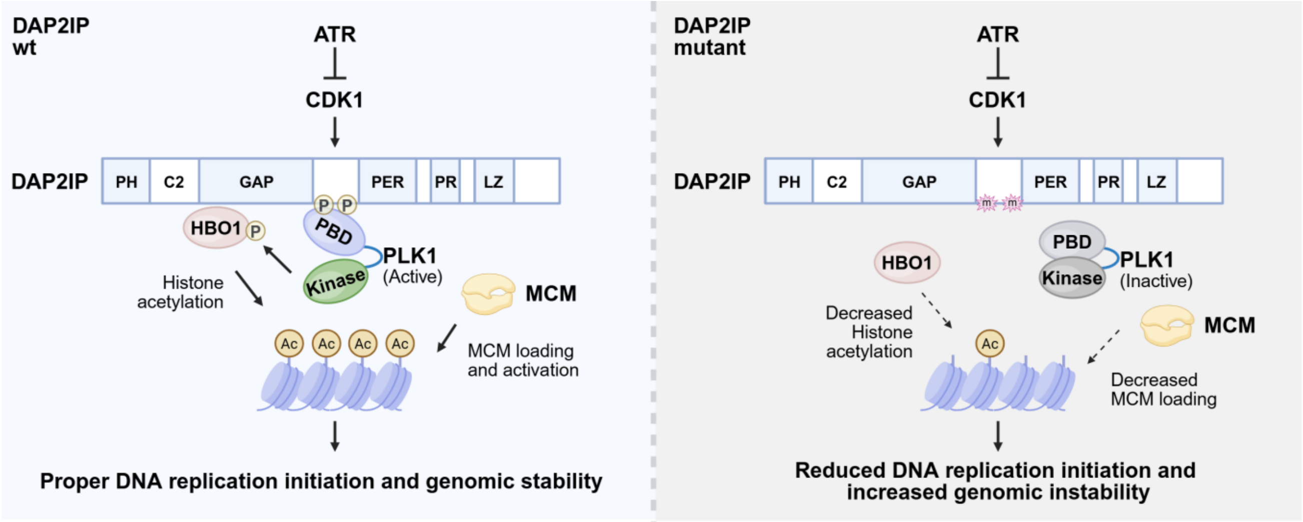

Figure S5. Proposed model role of DAB2IP in replication regulation and genomic stability maintenance. DAB2IP interacts with HBO1 through its GAP domain. Meanwhile, CDK1 phosphorylates DAB2IP on its Thr-531 and Thr-546 sites. These phosphorylation sites provide a docking position for PLK1 and activate the kinase activity of PLK1. PLK1 then phosphorylates HBO1 to promote the HBO1-mediated acetylation of histones and facilitate MCM chromatin loading and the onset of DNA replication. Loss of expression or CDK1-mediated phosphorylation of DAB2IP leads to incomplete replication of genomic DNA, resulting in genomic instability. ATR negatively regulates CDK1-mediated phosphorylation of DAB2IP to prevent excess DNA synthesis.
