# Supplementary material for: Nuclear DAB2IP regulates DNA replication initiation through activating PLK1-mediated HBO1 phosphorylation
Source: Nucleic Acids Res. 2025 Nov 20;53(21):gkaf1179. doi: 10.1093/nar/gkaf1179 (PMC12630137; doi:10.1093/nar/gkaf1179)

Figure 2C

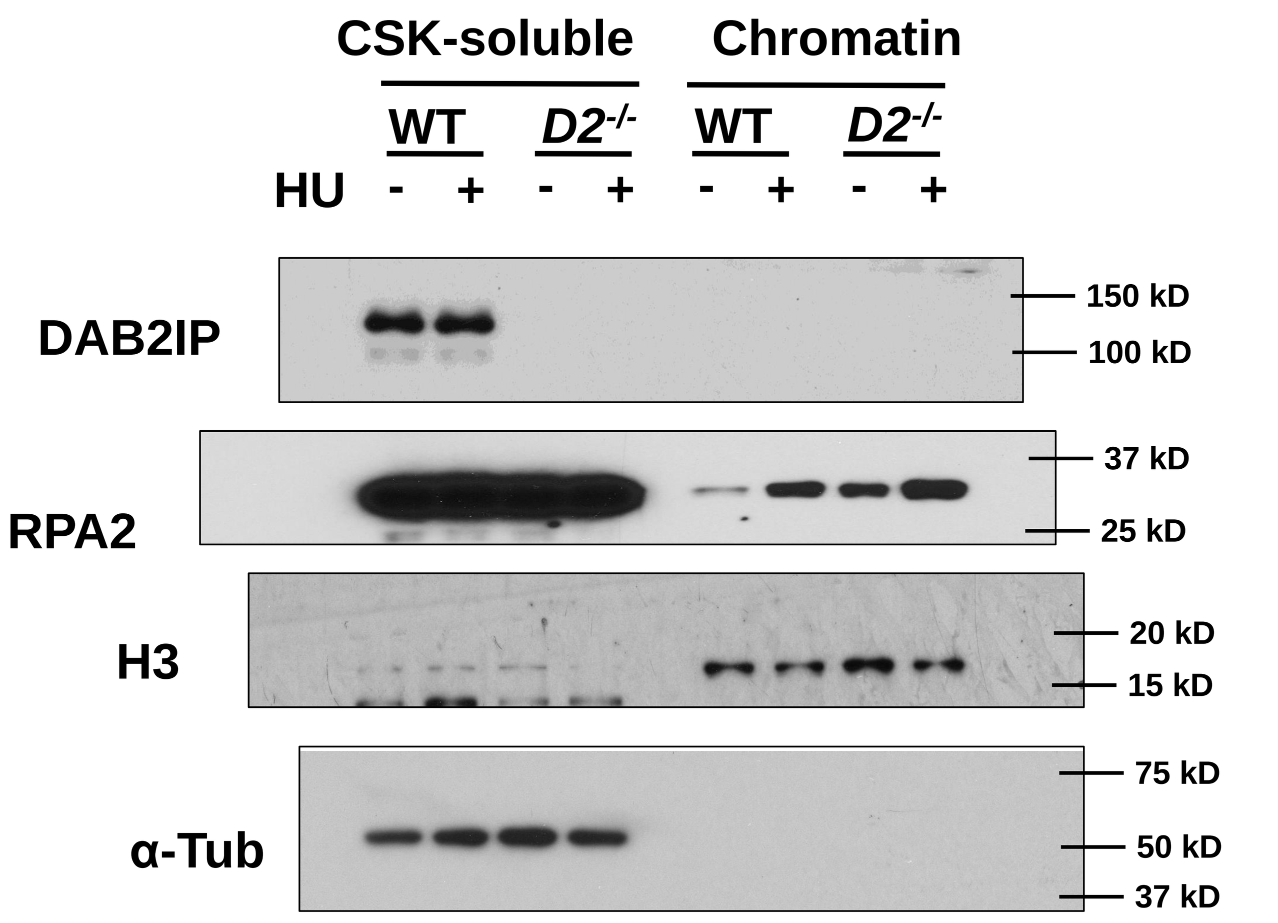

Figure 3A

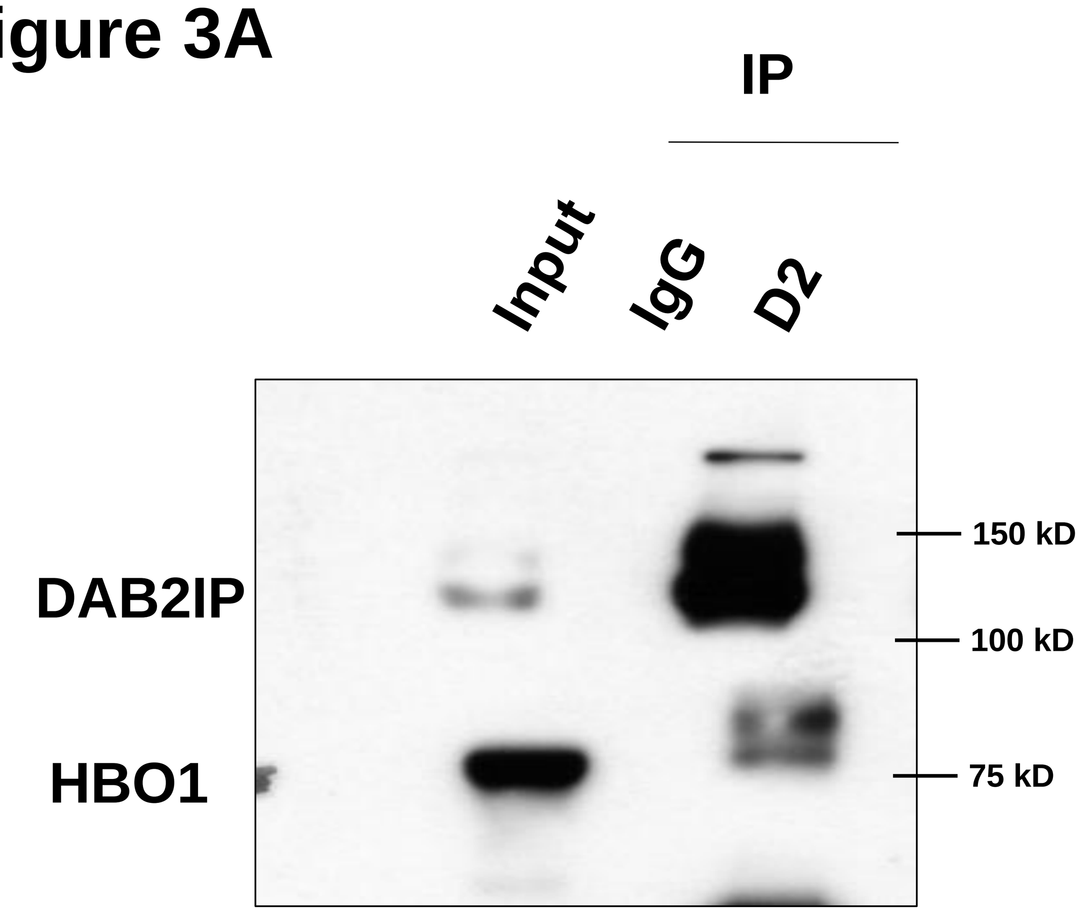

Figure 3B

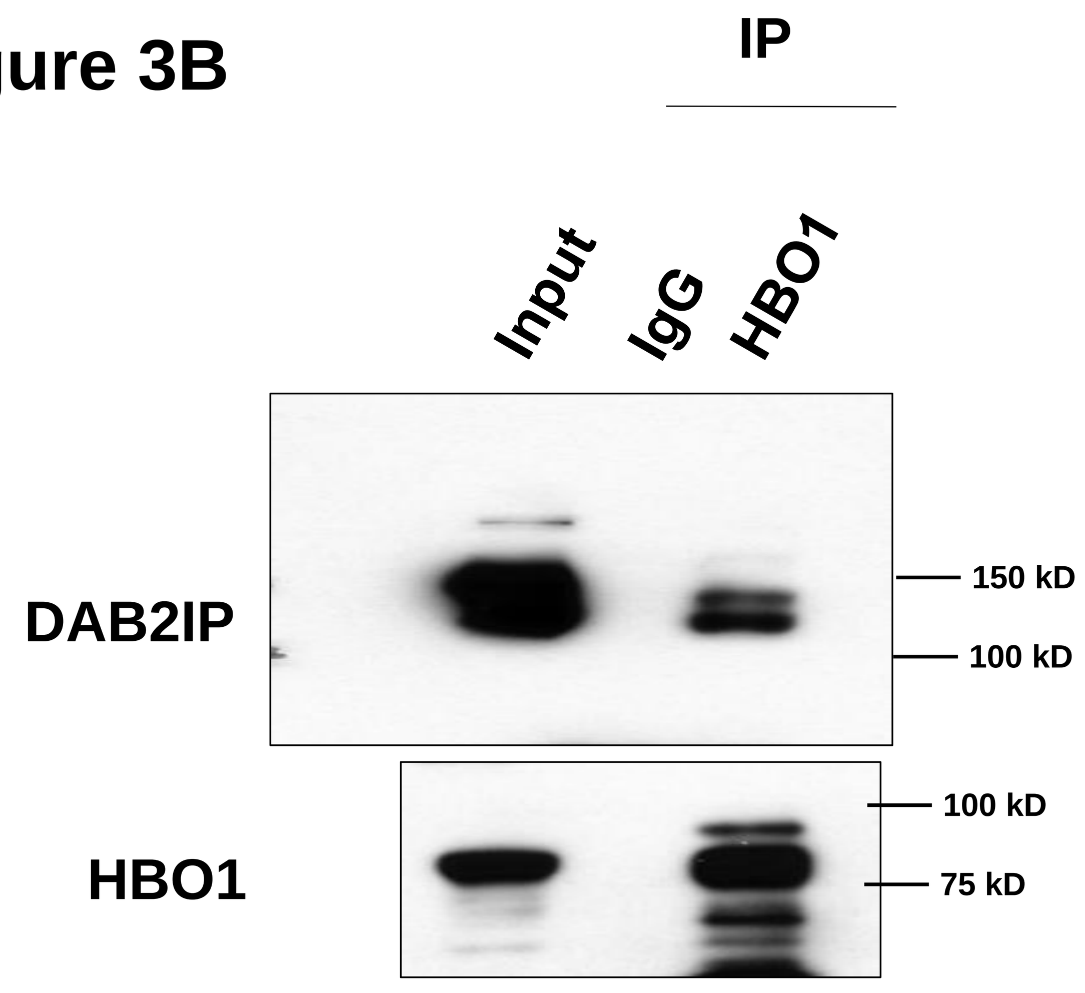

Figure 2F

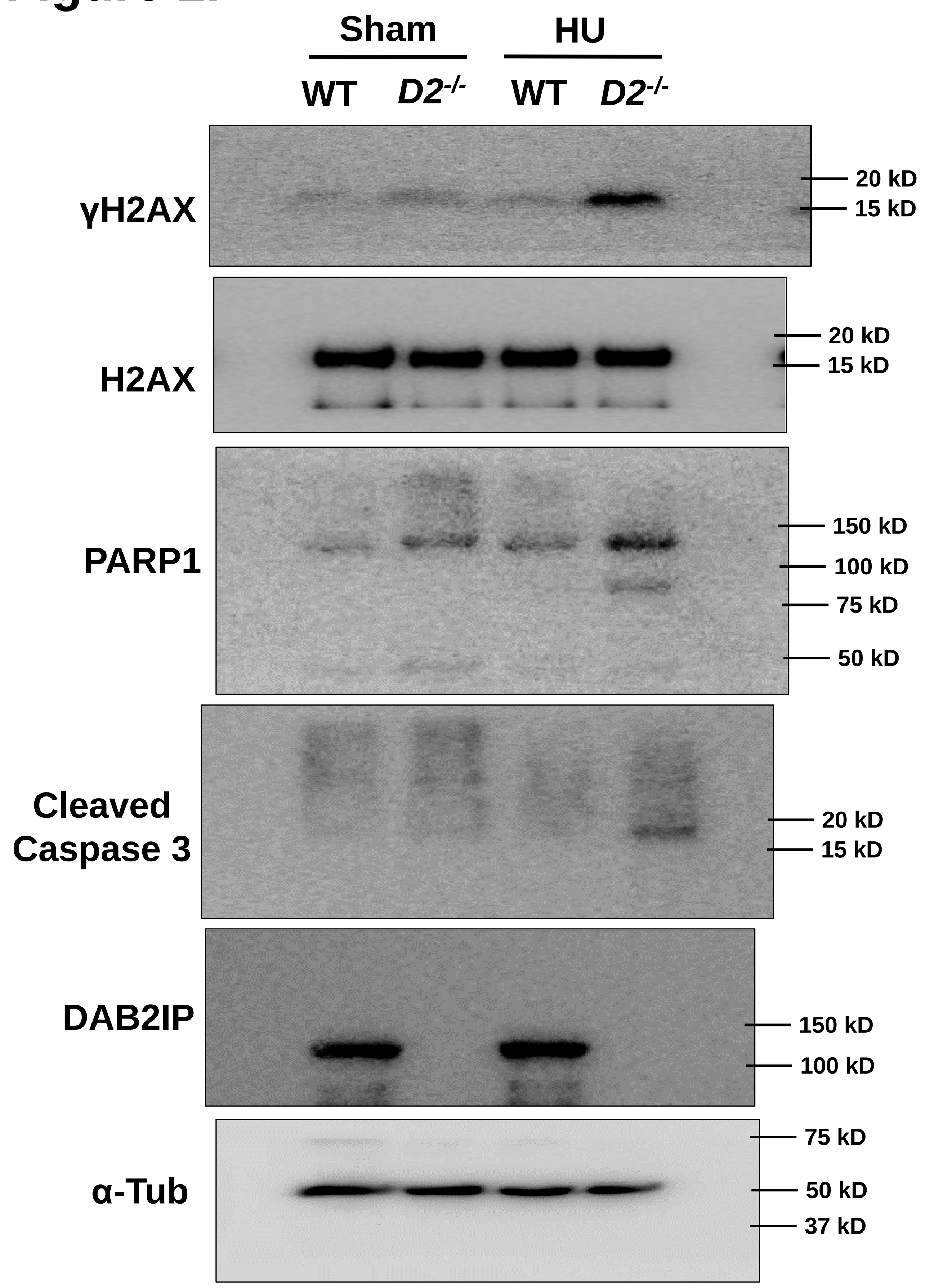

Figure 3D

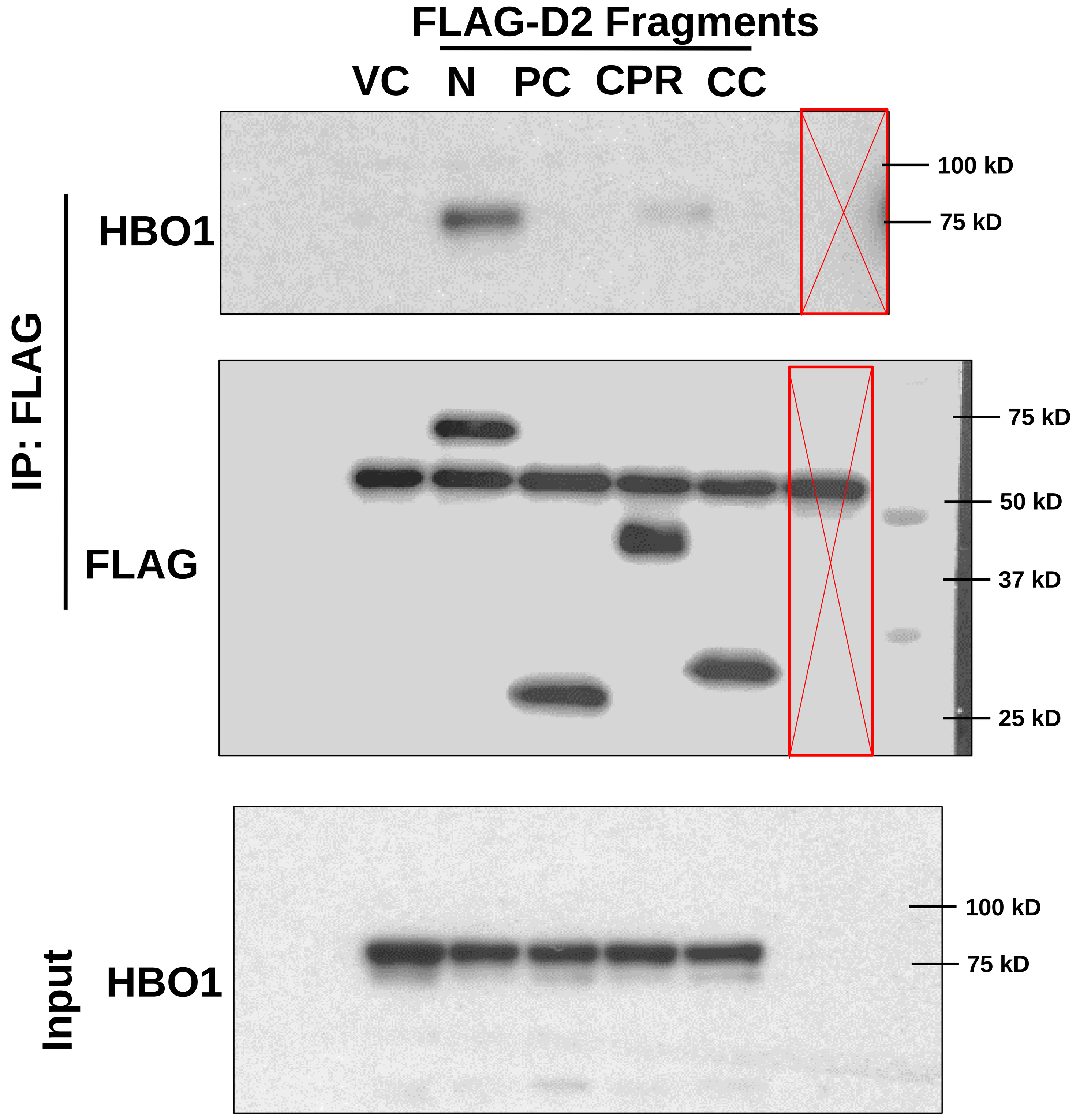

Figure 3G

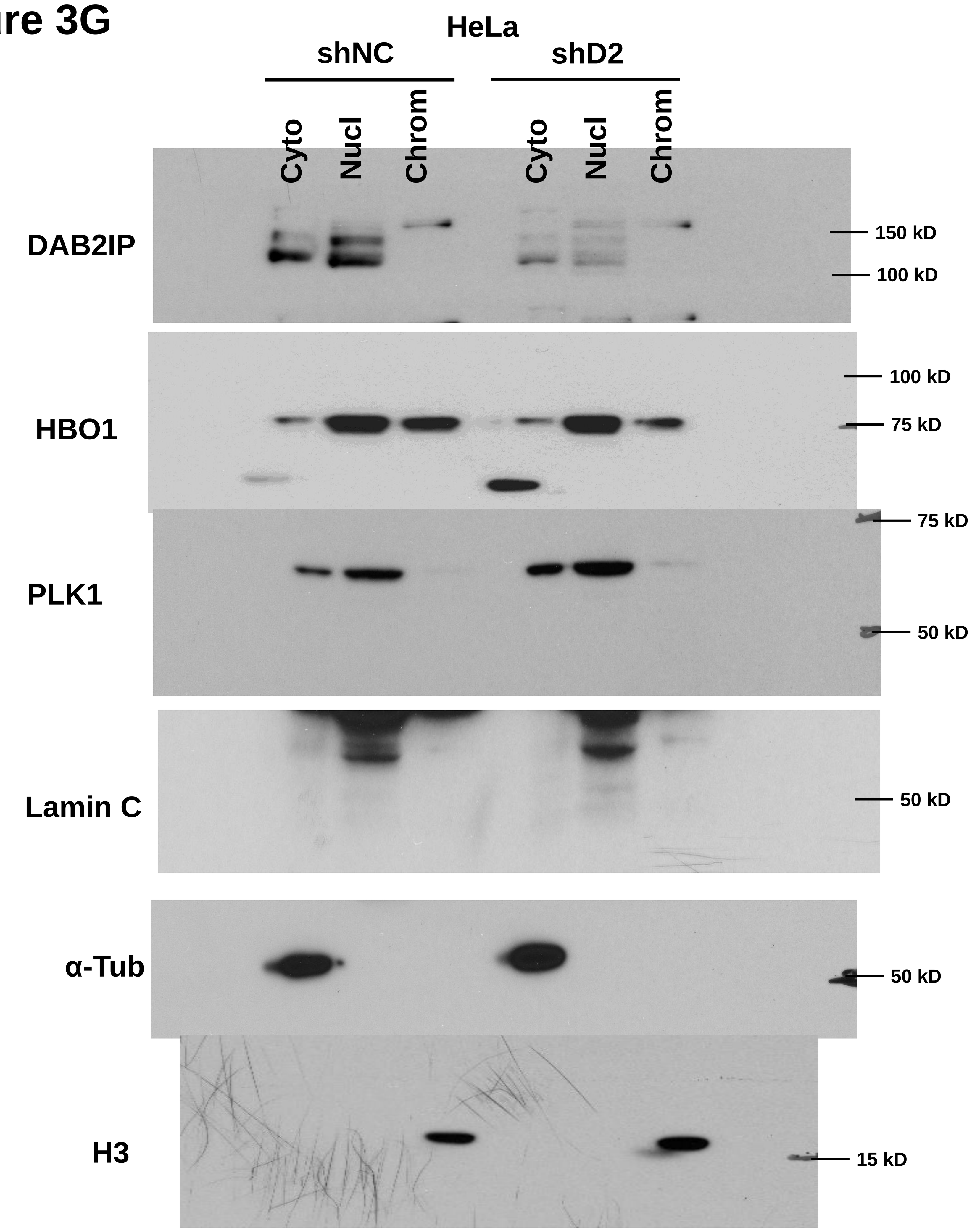

Figure 4A

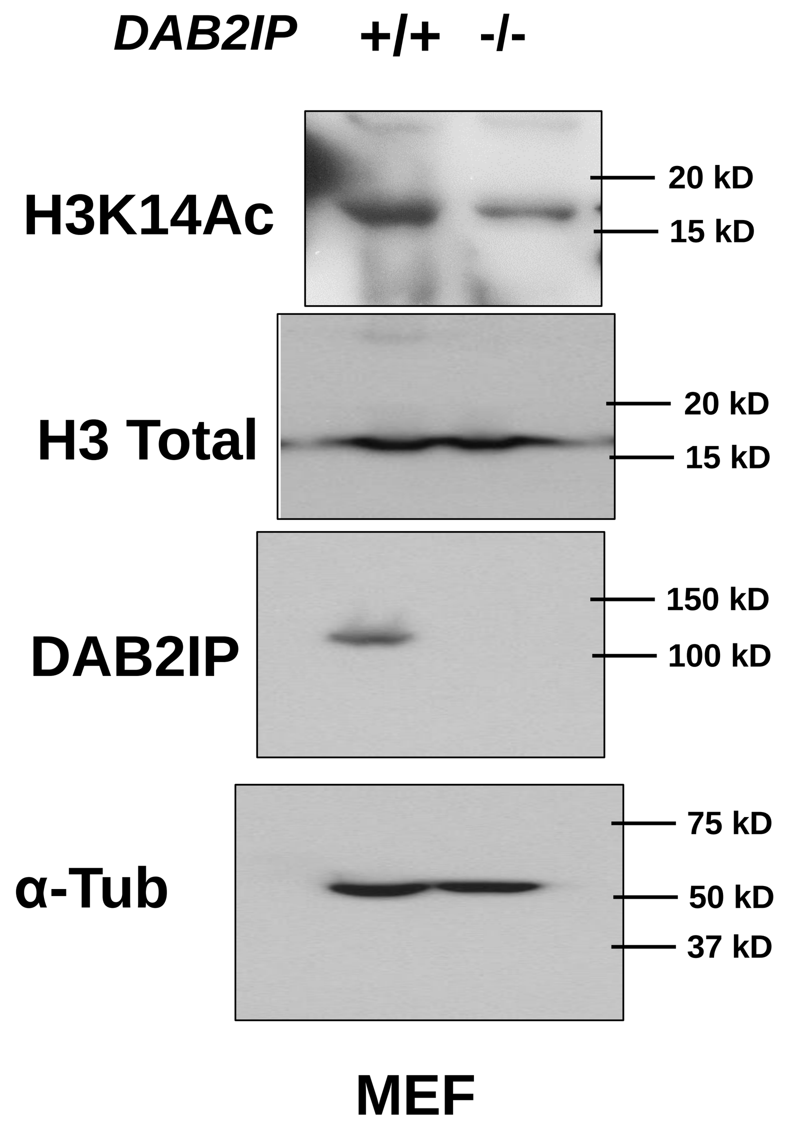

Figure 4D

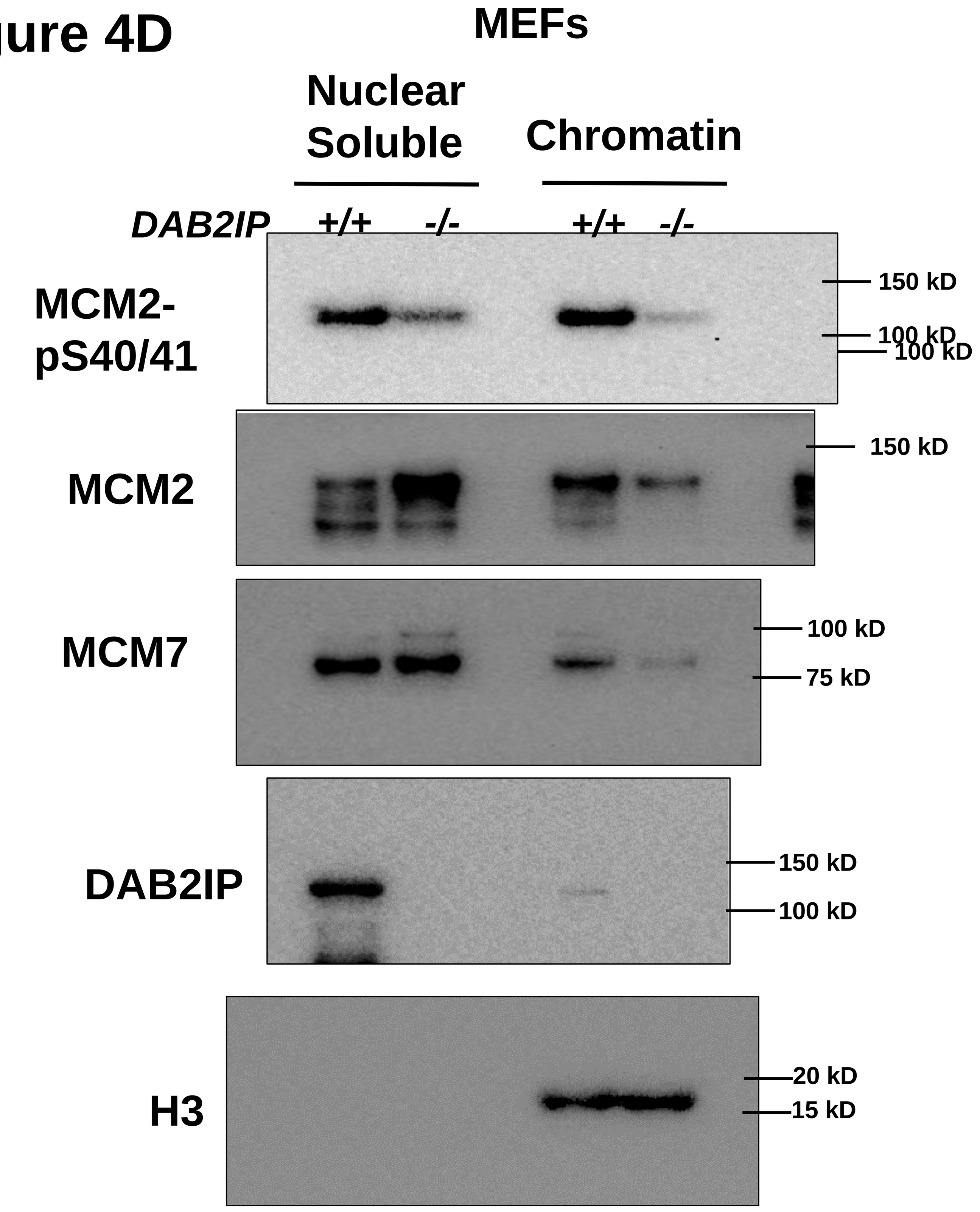

Figure 4E

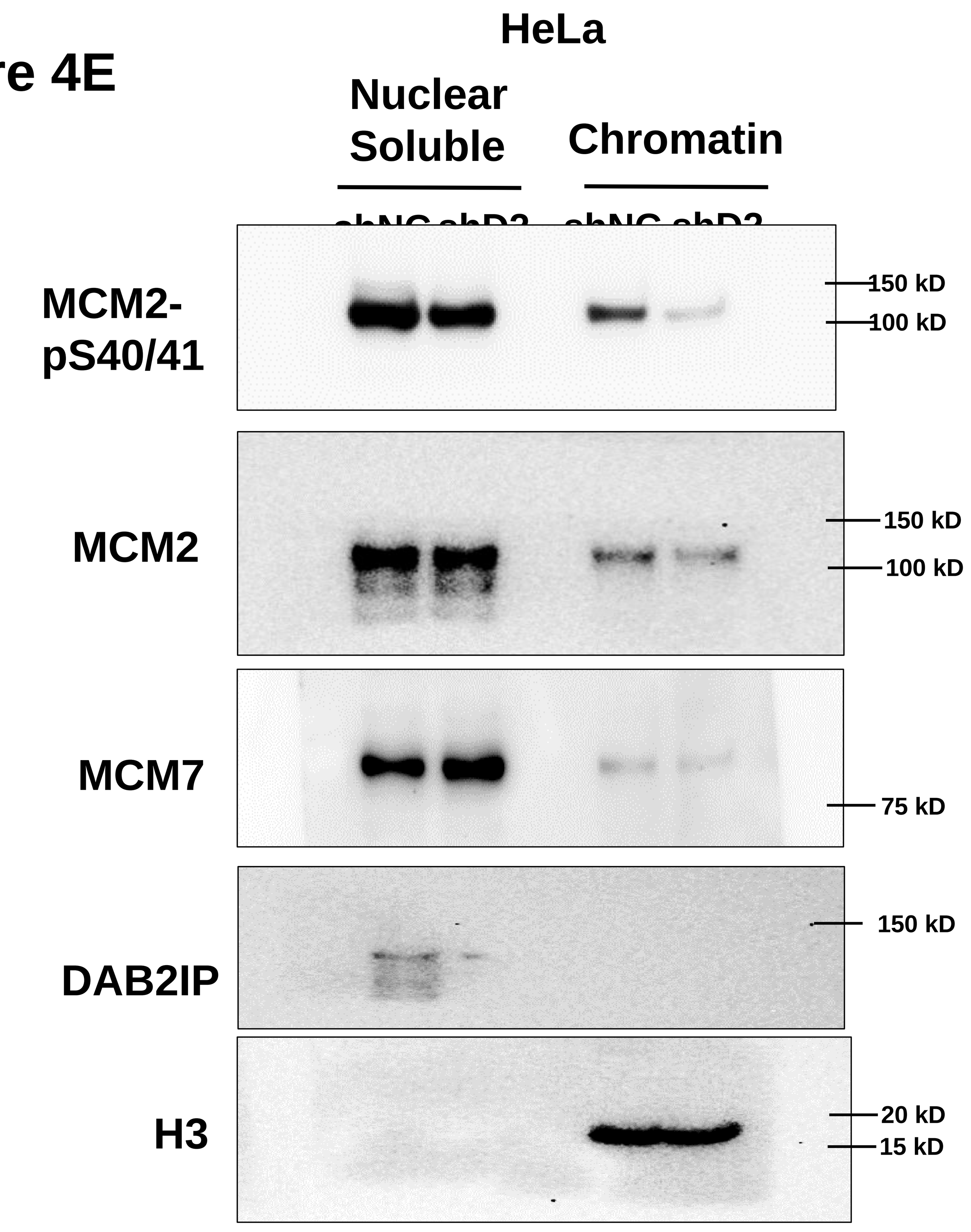

Figure 5A

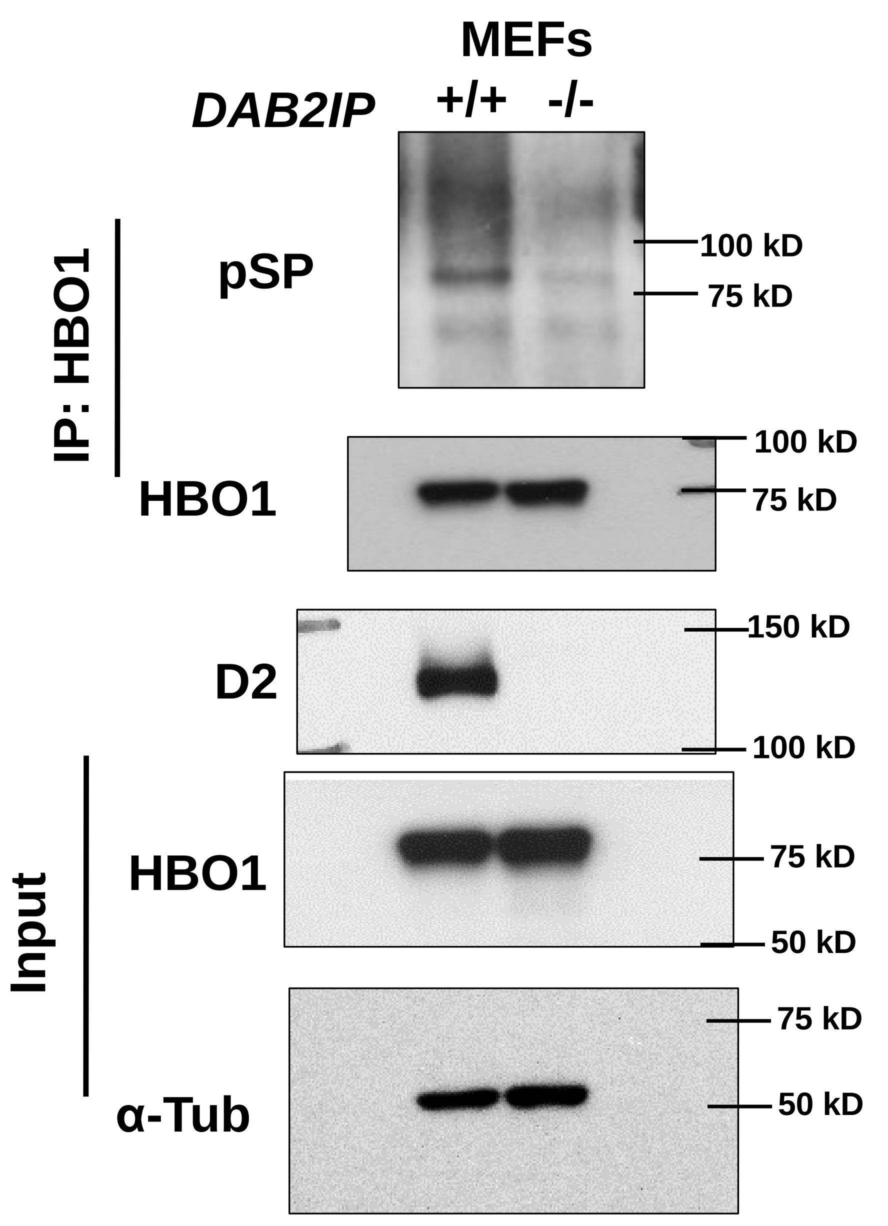

Figure 5B

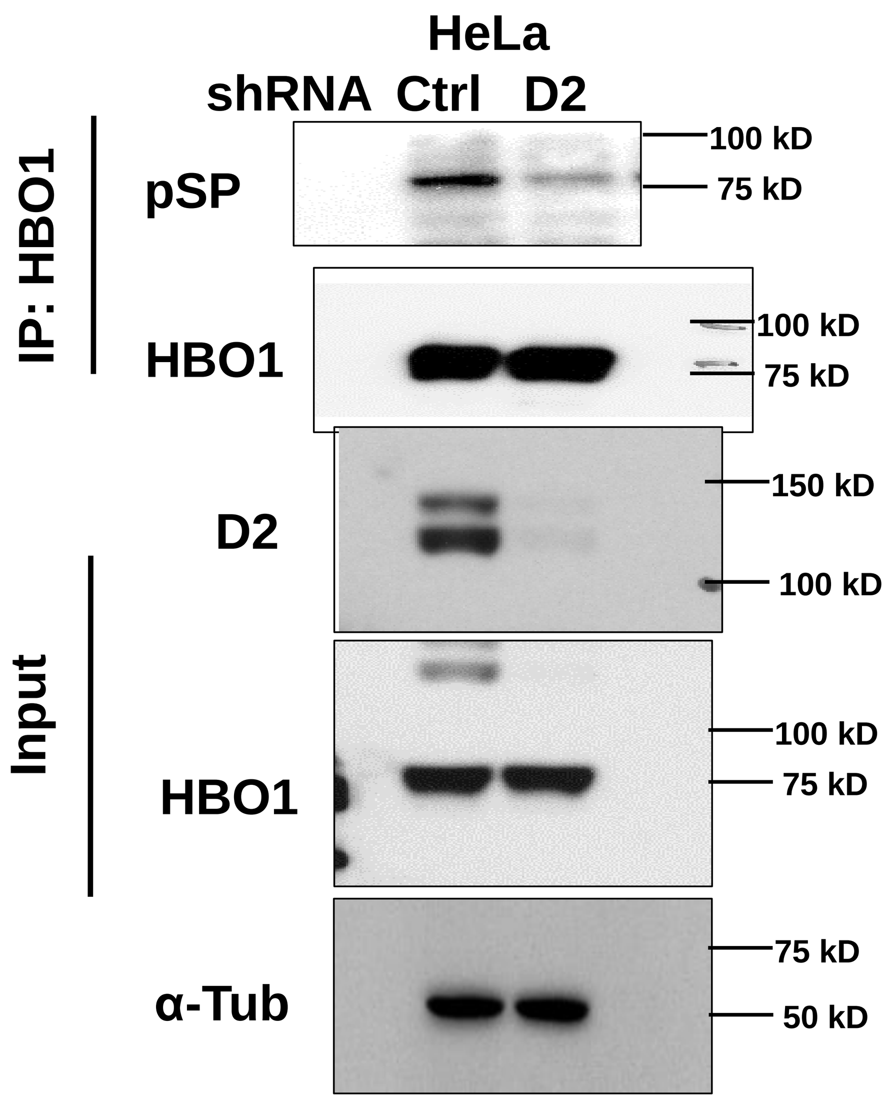

Figure 5C

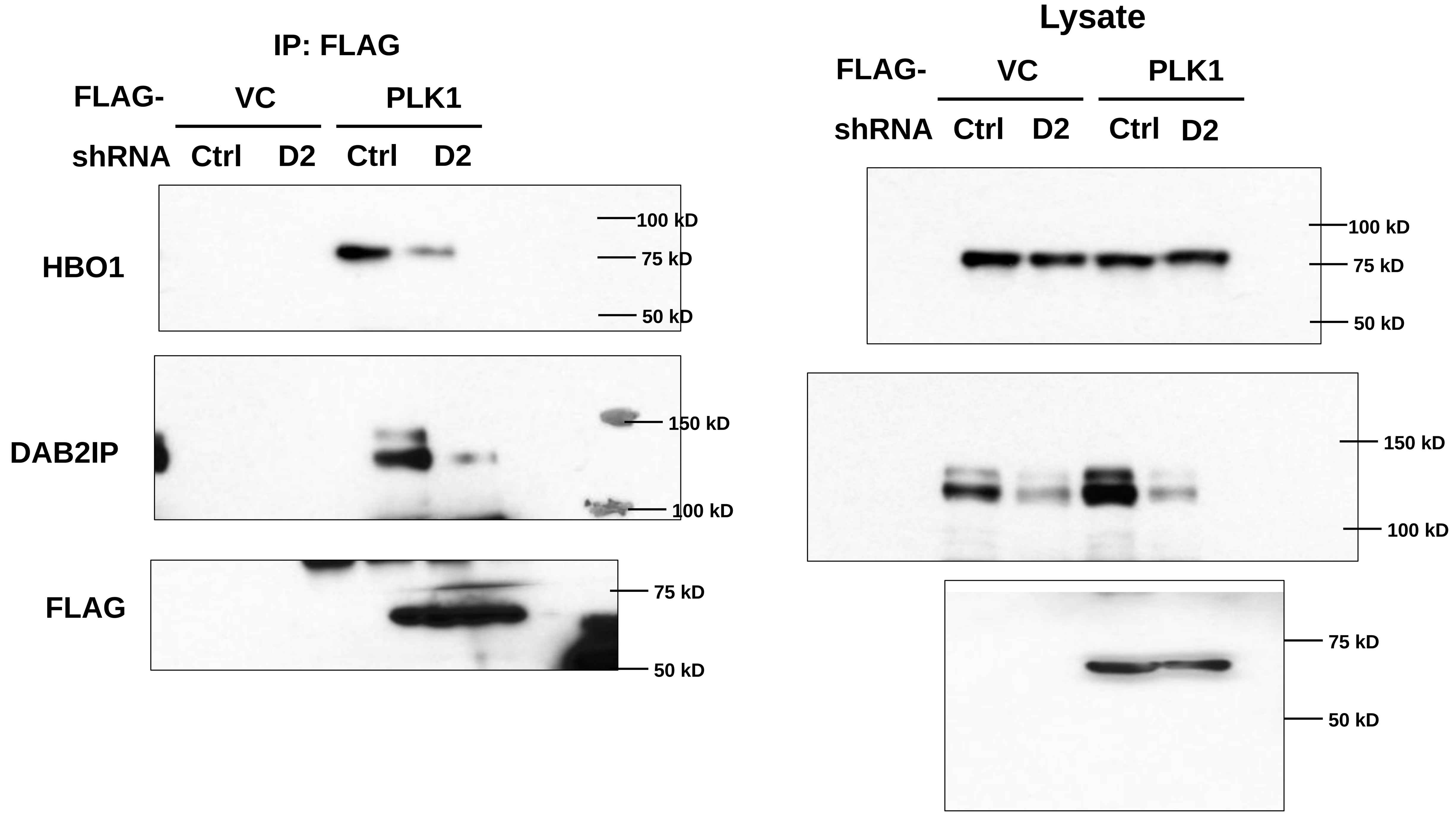

Figure 6A

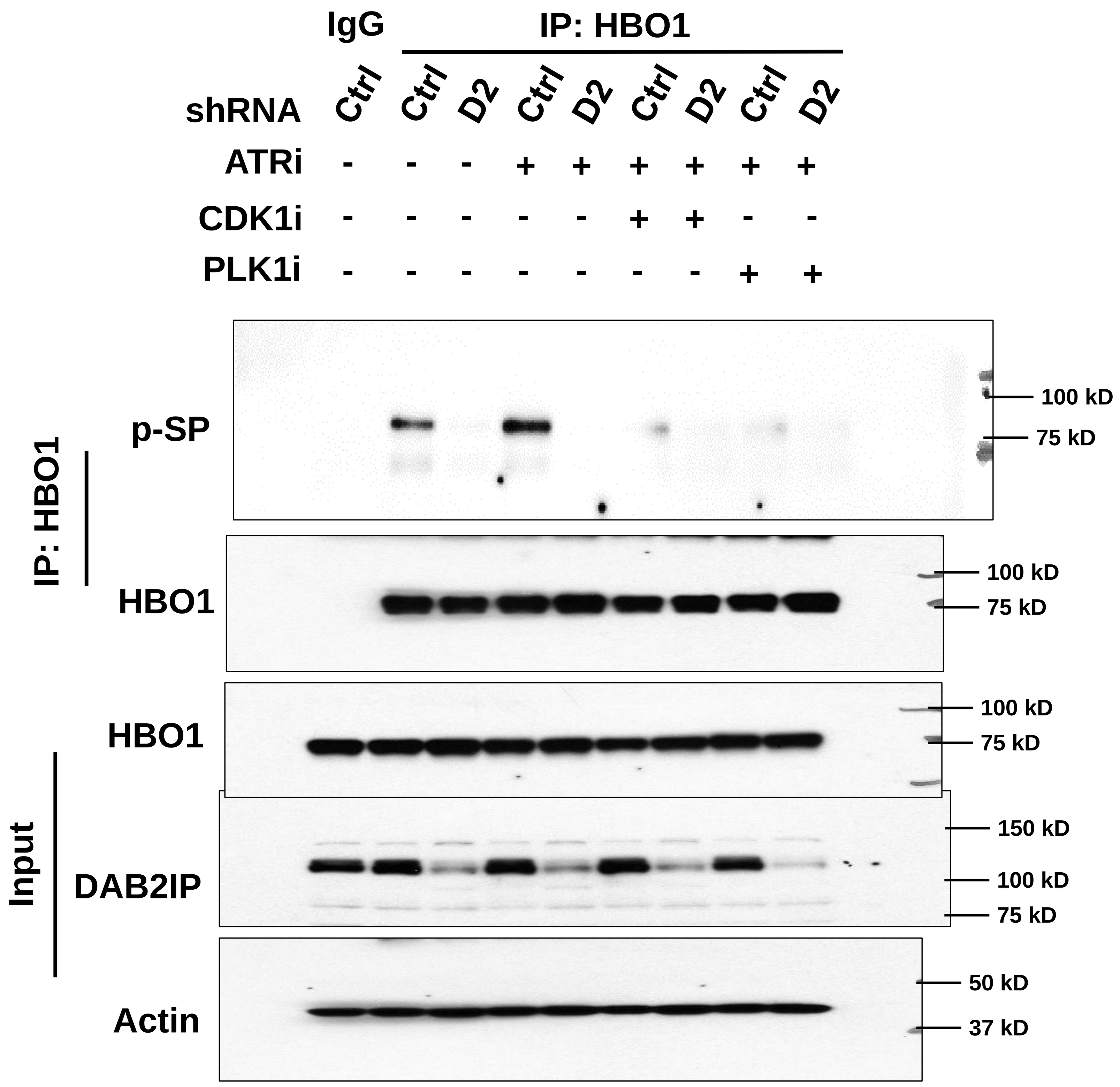

Figure 6B

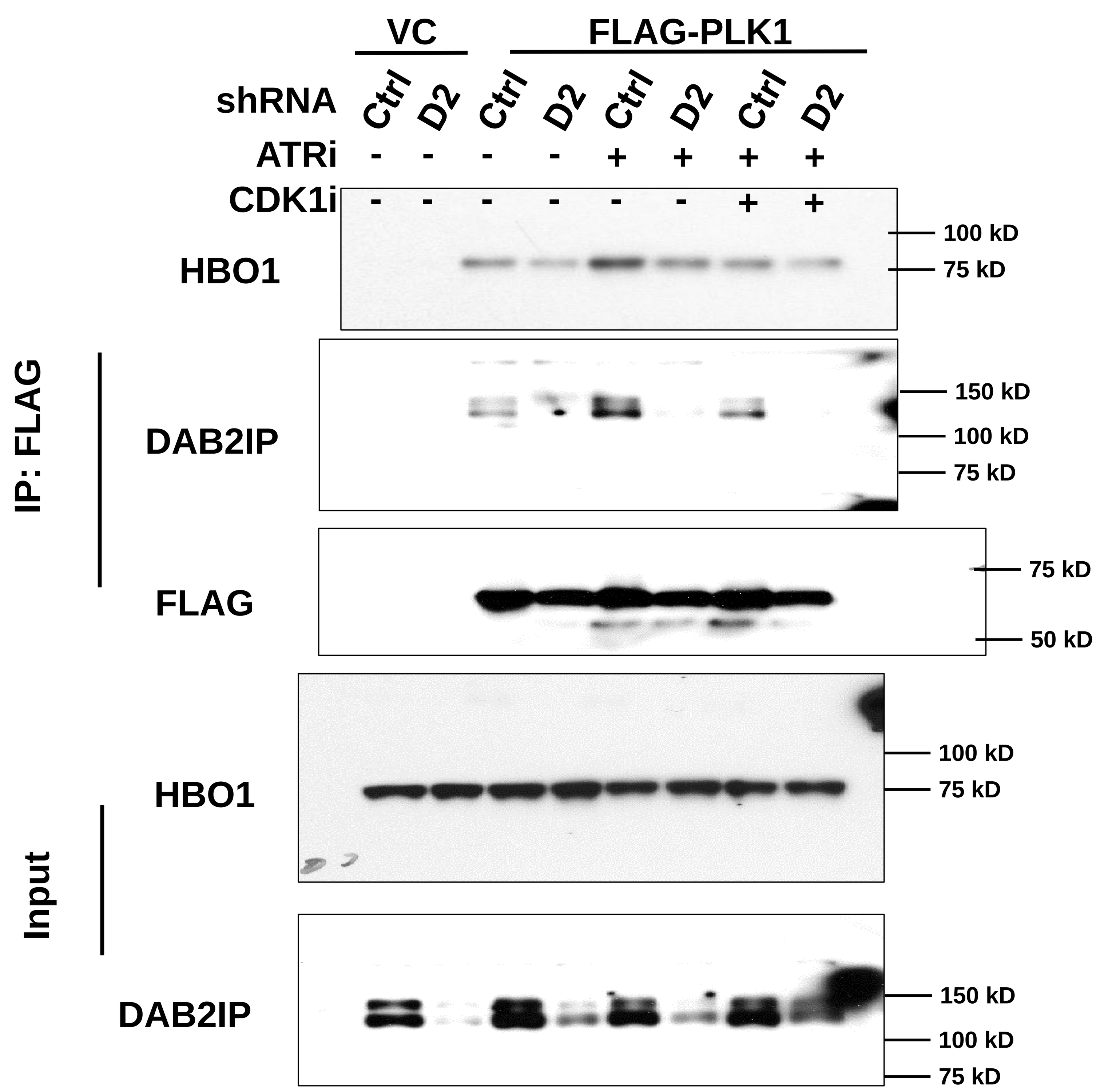

Figure 6E

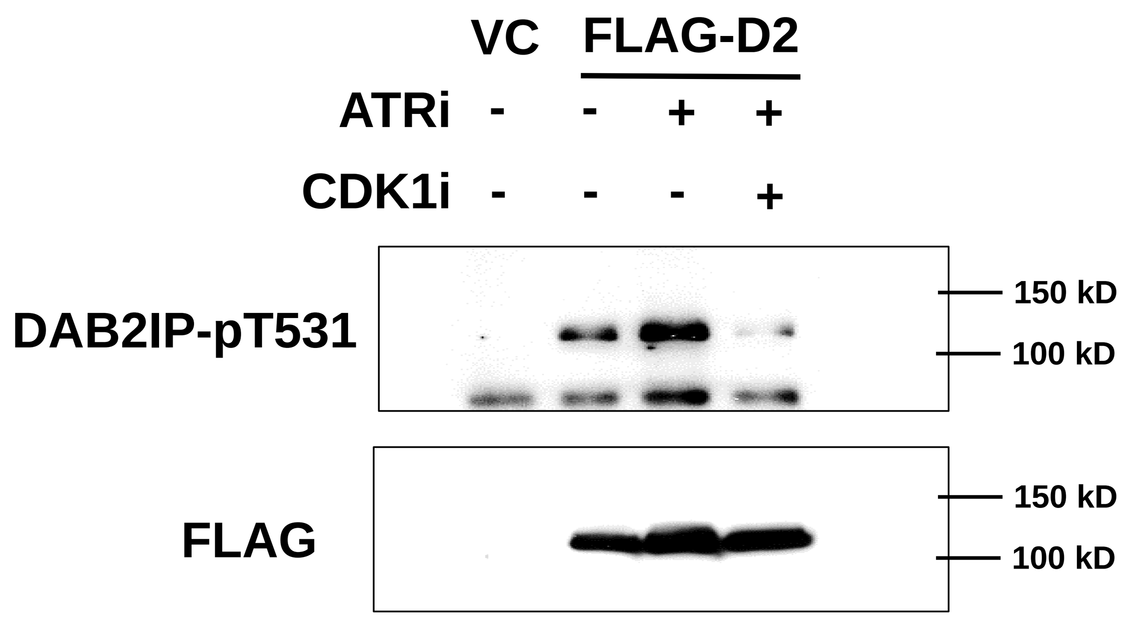

Figure 6F

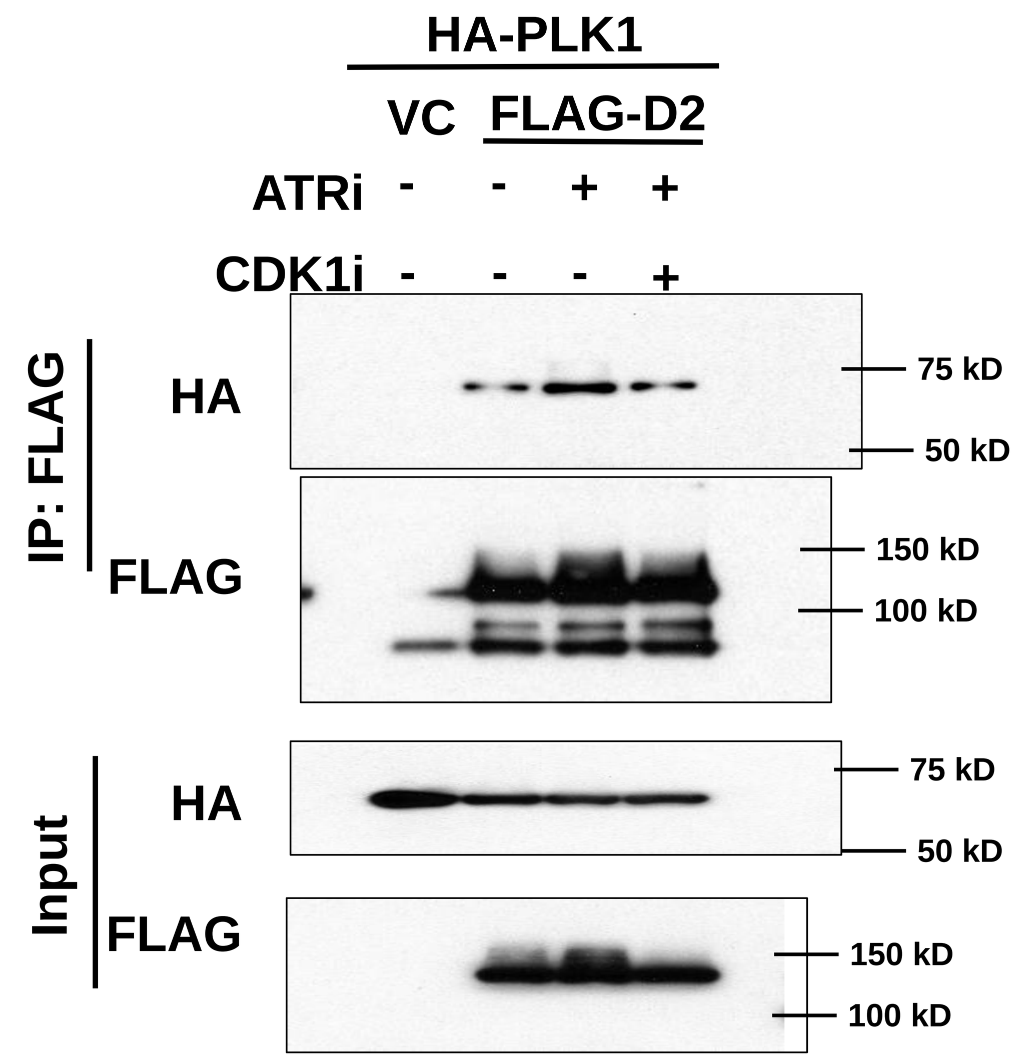

Figure 6G

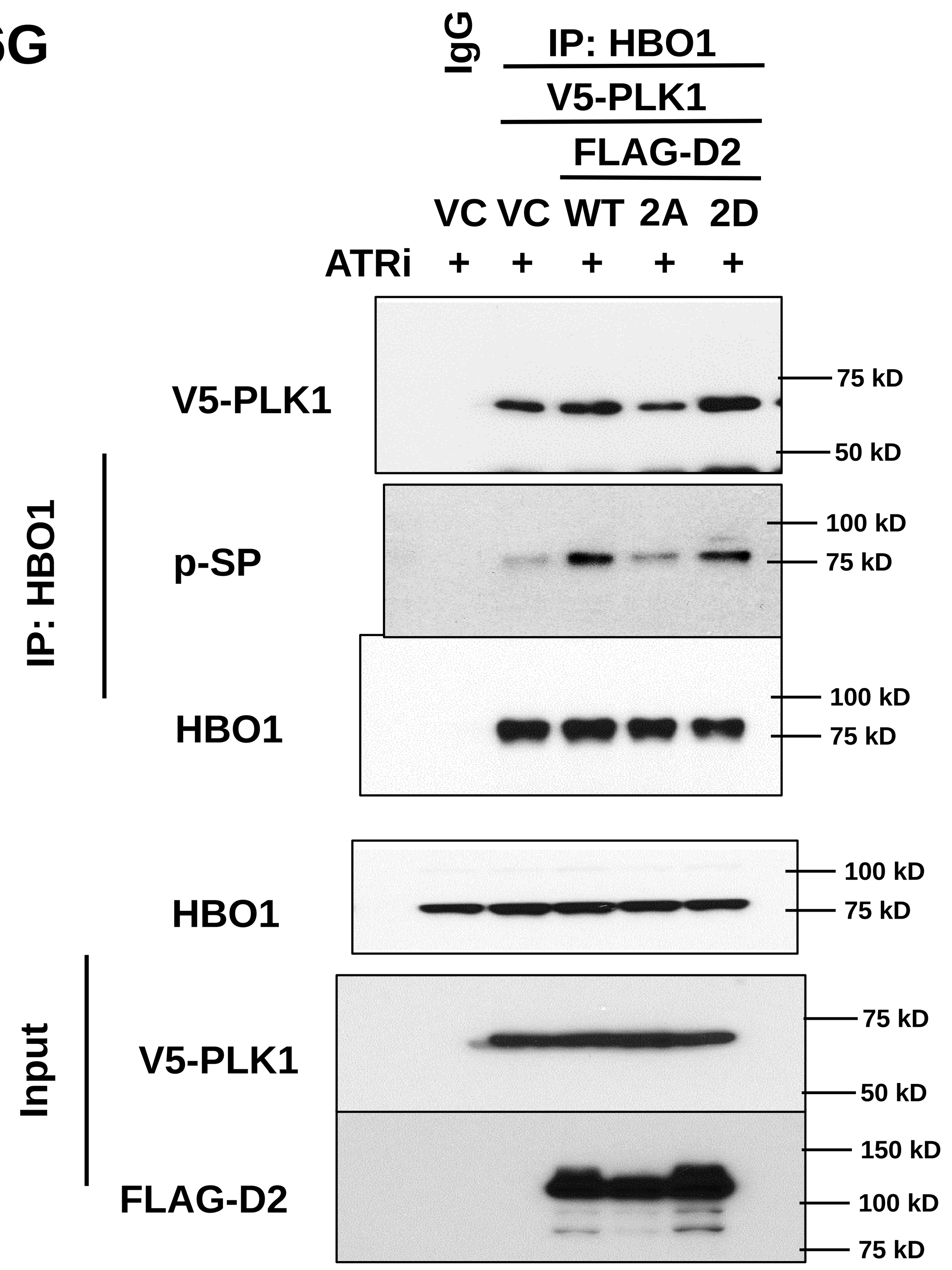

Figure 6H

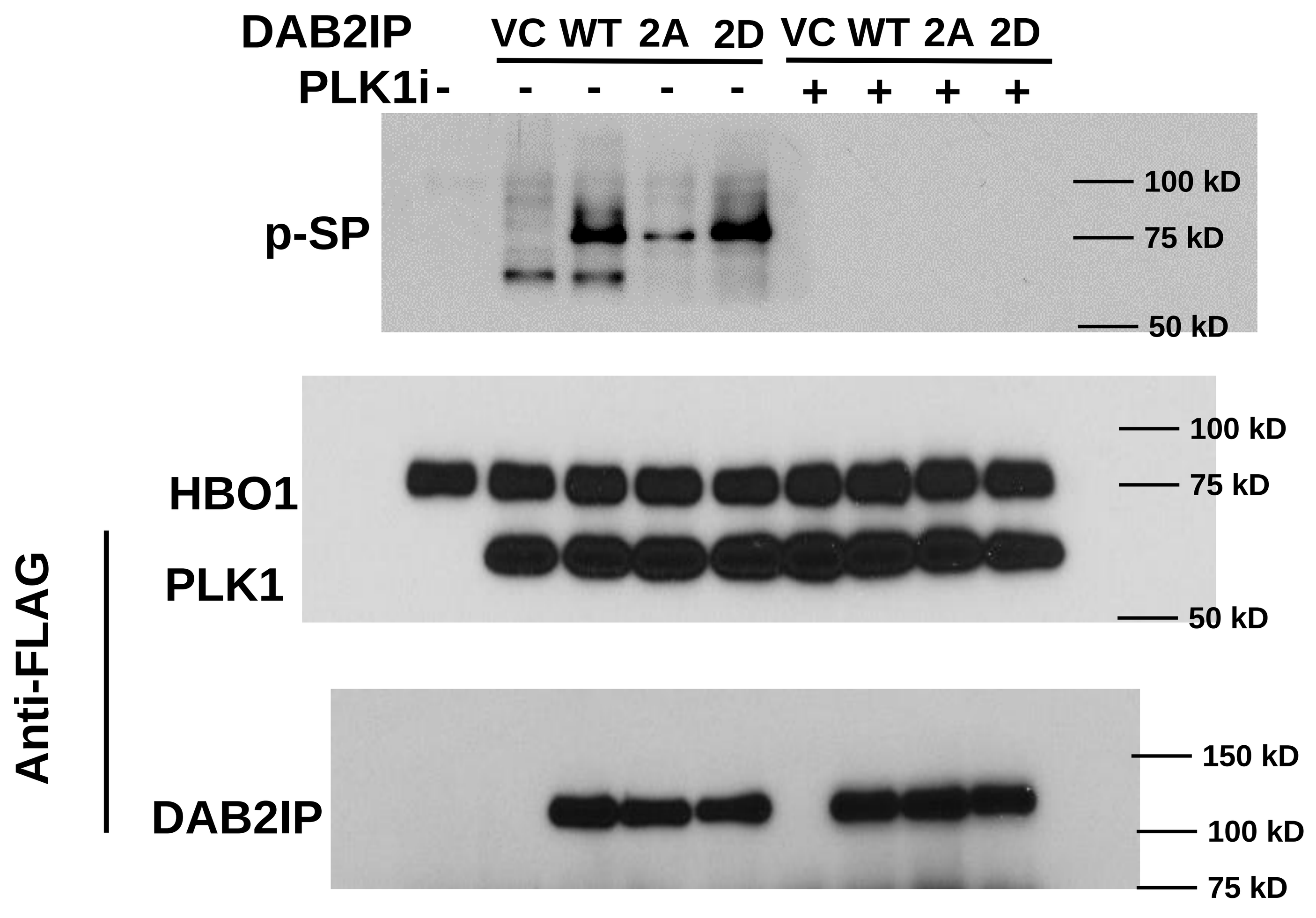

Supplement: gkaf1179_Supplemental_Files [file gkaf1179_supplemental_files.zip › Source data.zip!/Supplementary Information WB.pdf]
